# Effect of host movement on the prevalence of vector-borne diseases

**DOI:** 10.1007/s00285-025-02254-5

**Published:** 2025-09-05

**Authors:** Daozhou Gao, Yuan Lou

**Affiliations:** 1 Department of Mathematics and Statistics, Cleveland State University, Cleveland, OH 44115 USA; 2 School of Mathematical Sciences, CMA-Shanghai and MOE-LSC, Shanghai Jiao Tong University, Shanghai, 200240 China

**Keywords:** Human movement, Disease prevalence, Host reproduction number, Dispersal rate, Monotonicity, Disease persistence, 92D30, 34D05, 34C60, 34C12

## Abstract

Human movement plays a key role in spreading vector-borne diseases globally. Various spatial models of vector-borne diseases have been proposed and analyzed, mainly focusing on disease dynamics. In this paper, based on a multi-patch Ross–Macdonald model, we study the impact of host migration on the local and global host disease prevalences. Specifically, we find that the local disease prevalence of any patch is bounded by the minimum and maximum disease prevalences of all disconnected patches and establish a weak order-preserving property. For global disease prevalence, we derive its formula at both zero and infinite dispersal rates and compare them under certain conditions, and calculate the right derivative at no dispersal. In the case of two patches, we give two complete classifications of the model parameter space: one is to compare the host disease prevalences with and without host dispersal, and the other is to determine the monotonicity of host disease prevalence with respect to host dispersal rate. Numerical simulations confirm inconsistence between disease persistence and host disease prevalence, as well as between host prevalence and vector prevalence in response to host movement. In general, a more uneven distribution of hosts and vectors in a homogeneous environment leads to lower host prevalence but higher vector prevalence and stronger disease persistence.

## Introduction

Vector-borne diseases are illness transmitted to humans and animals by vectors such as mosquitoes, ticks, snails and bugs. These diseases, including malaria, Lyme disease, schistosomiasis and Chagas disease, pose a significant threat to global health. For example, the World Malaria Report 2024 reveals that there were approximately 263 million malaria cases and 597,000 malaria-related deaths over the globe in 2023 (World Health Organization 2024). Mathematical models of vector-borne diseases have long been useful in predicting spread trend and evaluating control strategies. The first vector-borne disease model, now known as the Ross–Macdonald model, was introduced to study the transmission of malaria by Ross in 1911 (Ross 1911) and subsequently refined by Macdonald in the 1950s (Macdonald 1957). Since then, this model has been extensively generalized to account for a wide range of biological, epidemiological, immunological, socioeconomic, and environmental factors, as well as intervention strategies (Koella 1991; Chitnis et al. 2006; Ruan et al. 2008; Okuneye and Gumel 2017; Jin et al. 2020), and adopted to analyze the transmission and control of other mosquito-borne or general vector-borne diseases (Velasco-Hernández 1991; Porco 1999; Yuan et al. 2021).

Population movement facilitates the geographic spread of vector-borne diseases. Infected travelers can introduce pathogens to non-endemic regions with suitable vector populations, triggering disease outbreaks in new regions. For instance, chikungunya, initially confined to Africa and Asia, was introduced to the Americas in 2013 and rapidly spread to most countries in the region within a year. Similarly, Zika infections were first detected in Brazil in March 2015, and subsequently spread to other parts of South and Central America, the Caribbean, and the United States. In a notable recent development, ten cases of locally acquired mosquito-transmitted malaria were reported in the United States during May-September 2023, making the first such cases in 20 years (Courtney et al. 2024). A deeper understanding of how population movement influences the spread of vector-borne diseases is essential for designing effective disease prevention and control measures.

Modern transportation enables the fast movement of people from one place to another, so patch models are widely used to describe disease spread in discrete space. Auger et al. (2008) generalized the Ross–Macdonald model from a single patch to *n* patches with host migration between patches and proved the global asymptotic stability of either the disease-free equilibrium or the endemic equilibrium. Cosner et al. (2009) proposed two classes of multi-patch Ross–Macdonald models incorporating host and vector movements, using Lagrangian (mimic daily commuting) and Eulerian (mimic migration) approaches. They obtained global dynamic results for both approaches and numerically demonstrated that human movement between two patches can shift a disease from extinction to persistence. This phenomenon was systematically explored by Gao and collaborators (Gao et al. 2019; Gao and Cao 2024; Gao and Yuan 2024), who showed that nonhomogeneous mixing of hosts and vectors in a homogeneous environment promotes disease persistence measured by the basic reproduction number. Arino et al. (2012) constructed a metapopulation malaria model with host migration and partial community. The basic reproduction number $\mathcal {R}_0$ was shown to govern the local stability of the disease-free equilibrium and the model system can exhibit a backward bifurcation at $\mathcal {R}_0=1$. Gao and Ruan (2012) formulated a multi-patch malaria model with SEIRS and SEI structures for hosts and vectors, respectively. They found that travel can promote or inhibit disease persistence even between two identical patches. Other generalizations of the Ross–Macdonald model in a patchy environment include seasonality (Gao et al. 2014), multiple strains (Qiu et al. 2013), latencies in hosts and vectors (Xiao and Zou 2014) and so on. For Lagrangian and reaction-diffusion models of vector-borne diseases, interested readers are directed to the works of Hasibeder and Dye (1988), Bichara and Castillo-Chavez (2016), Gao and Cao (2024), Lou and Zhao (2010), Magal et al. (2018) and the references therein.

Eradicating an infectious disease is rarely achievable due to a complex interplay of biological, ecological, socioeconomic, and logistical factors. The World Health Organization (WHO) launched the Global Malaria Eradication Program (GMEP) in 1955, but the campaign was discontinued in 1969 when it became evident that eradication was unfeasible. Today, malaria remains endemic in 83 countries. Consequently, reducing disease prevalence—the proportion of people being infected—to a level where the disease no longer poses a major public health threat is a more practical and beneficial goal. While the basic reproduction number $\mathcal {R}_0$ often determines whether a disease can spread across space, it usually cannot measure disease prevalence when the disease is endemic. Since epidemic patch models are typically in the form of high-dimensional nonlinear systems, theoretical studies on disease prevalence are extremely rare. Until recently, based on an SIS patch model for directly transmitted diseases like common cold and gonorrhea, we explored the influence of human movement on the disease prevalence within an individual patch and across all interconnected patches (Gao 2020; Gao and Lou 2021; Gao and Wang 2024).

This paper aims to examine the impact of host dispersal on the local and global disease prevalence among hosts. In Sect. 2, we formulate a multi-patch Ross–Macdonald model with host migration and present some preliminaries on the basic reproduction number and disease dynamics. In Sect. 3, for the *n*-patch model, the local disease prevalence is estimated and a weak order preserving property is established. Additionally, the global disease prevalences are calculated at zero and infinite dispersal rates and compared under specific conditions, and the right derivative of the global disease prevalence at zero dispersal is evaluated. Section 4 focuses on the two-patch submodel, offering two complete classifications on the global host disease prevalence. In Sect. 5, numerical simulations are conducted to further reveal the influence of host dispersal on both host and vector disease prevalences. Finally, we conclude with a discussion highlighting the novelty and limitations of the study.

## The model

We aim to investigate the spread of a vector-borne disease, such as malaria, as it propagates through host migration within a discrete space composed of $n\ge 2$ patches. The movement of vectors is ignored because of their limited mobility. In patch $i\in \Omega =\{1,\dots ,n\}$, both hosts and vectors are classified as either susceptible or infected, with their total population sizes denoted by $H_i$ and $V_i$, respectively. We consider a multi-patch Ross–Macdonald model with nonnegative initial condition as follows:

2.1 $$\begin{aligned} \begin{aligned} \dfrac{dH_i}{dt}&=\varepsilon \sum \limits _{j\in \Omega }L_{ij}H_j,\quad i\in \Omega , \\ \dfrac{dh_i}{dt}&=a_ib_i\frac{v_i}{H_i}(H_i-h_i)-\gamma _i h_i+\varepsilon \sum \limits _{j\in \Omega }L_{ij}h_j,\quad i\in \Omega , \\ \dfrac{dv_i}{dt}&=a_ic_i\frac{h_i}{H_i}(V_i-v_i)-\mu _i v_i,\quad i\in \Omega , \end{aligned} \end{aligned}$$

where $h_i$ and $v_i$ denote the number of infected hosts and infected vectors in patch *i*, respectively; $a_i$ is the mosquito biting rate in patch *i*; $b_i$ and $c_i$ are the probabilities of disease transmission per bite from an infected vector to a susceptible host and from an infected host to a susceptible vector in patch *i*, respectively; $\gamma _i$ and $\mu _i$ are the host recovery rate and vector mortality rate in patch *i*, respectively; $L_{ij}$ is the degree of incoming movement from patch *j* to patch *i* for $i \not = j$, and $-L_{ii}=\sum _{j\ne i}L_{ji}$ is the degree of outgoing movement from patch *i* to all other patches; and $\varepsilon$ is the host dispersal rate. Similar models have been proposed by Auger et al. (2008) and Cosner et al. (2009), who also derived the global dynamic results. When no host movement, i.e., $\varepsilon =0$, the model of each patch is exactly the classical Ross–Macdonald model (Jin et al. 2020; Ross 1911; Macdonald 1957).

Unless otherwise indicated, all parameters except these involved in the connectivity matrix $L=(L_{ij})_{n\times n}$ are assumed to be positive constants. In addition, we make the following assumptions throughout this paper:

1. Either $h_i(0)>0$ or $v_i(0)>0$, and $H_i(0)>0$ and $V_i(0)>0$ for all $i\in \Omega$.
2. *L* is essentially nonnegative (or say quasi-positive if all off-diagonal entries are nonnegative) and irreducible.
3. The host population distribution across patches at no dispersal obeys the limiting case of $\varepsilon \rightarrow 0+$.

The model (2.1) admits a unique disease-free equilibrium *E*_0_ = (H^*^, ***h***^0^, ***v***^0^) = ($$H^*_1$$ …, $$H^*_n,$$ 0, …, 0, 0, …, 0), where H^*^ = $$(H^*_1$$ …, $$H^*_n),$$ is the unique positive solution to the host migration model, namely

$$\begin{aligned} \begin{aligned} \sum \limits _{j\in \Omega } L_{ij}H_j=0,\ \ i\in \Omega \ \ \text{ and } \ \ \sum \limits _{i\in \Omega }H_i=\sum _{i\in \Omega }H_i(0):=H. \end{aligned} \end{aligned}$$

In other words, $(H^*_1,\dots ,H^*_n)^{\mathop {{\textrm{T}}}}$ is the positive right eigenvector of *L* associated with the zero eigenvalue, normalized by *H*, and independent of $\varepsilon$. More precisely, it follows from Lemma 3.1 in Gao and Dong (2020) that

$$\begin{aligned} \begin{aligned} (H^*_1,\dots ,H^*_n)=\frac{H}{\sum _{k\in \Omega }L_{kk}^*}(L_{11}^*,\dots ,L_{nn}^*) :=H(\alpha _1,\dots ,\alpha _n), \end{aligned} \end{aligned}$$

where *L*_ii_^*^ is the (*i*, *i*)-cofactor of *L*, and *α*_*i*_ = *L*_ii_^*^/$\sum _{k\in \Omega }L_{kk}^*\in (0,1)$ with $\sum _{i\in \Omega }\alpha _i$ = 1. Since $H_i(t)\rightarrow H_i^*$ as $t\rightarrow \infty$, replacing *H*_*i*_ by *H*_i_^*^ in (2.1) yields the limiting system

2.2 $$\begin{aligned} \begin{aligned}&\dfrac{dh_i}{dt}=a_ib_i\frac{v_i}{H_i^*}(H_i^*-h_i)-\gamma _i h_i+\varepsilon \sum \limits _{j\in \Omega }L_{ij}h_j,\quad i\in \Omega , \\&\dfrac{dv_i}{dt}=a_ic_i\frac{h_i}{H_i^*}(V_i-v_i)-\mu _i v_i,\quad i\in \Omega , \end{aligned} \end{aligned}$$

which is topologically equivalent to the full system by the theory of asymptotically autonomous systems or chain transitive sets (Castillo-Chavez and Thieme 1995; Zhao 2017).

Following the next generation matrix method (Diekmann et al. 1990; van den Driessche and Watmough 2002), the basic reproduction number of model (2.1) is given by

2.3 $$\begin{aligned} \begin{aligned} \mathcal {R}_0=\rho \left( \begin{pmatrix} 0 & \quad \mathscr {A} \\ 0 & \quad 0 \end{pmatrix}\begin{pmatrix} \mathscr {C} & \quad 0 \\ \mathscr {B} & \quad \mathscr {D} \end{pmatrix}^{-1}\right) =\rho (\mathscr {A}{\mathscr {D}}^{-1}\mathscr {B}{\mathscr {C}}^{-1}), \end{aligned} \end{aligned}$$

where

$$\mathscr {A}=\mathop {{\textrm{diag}}}(a_ib_i),\ \ \mathscr {B}=\mathop {{\textrm{diag}}}(a_ic_iV_i/H^*_i),\ \ \mathscr {C}=\mathop {{\textrm{diag}}}(\gamma _i)-\varepsilon L,\ \ \text{ and } \ \ \mathscr {D}=\mathop {{\textrm{diag}}}(\mu _i).$$

In the above derivation, to emphasize host infections, we treat only host infections as new infections and vector infections as transitions, consistent with Brauer et al. (2019) and Yuan et al. (2021). If otherwise, the basic reproduction number would take a square root form, as seen in Auger et al. (2008) and Cosner et al. (2009). In particular, the basic reproduction number of patch *i* in disconnection ($\varepsilon =0$) is

$$\begin{aligned} \mathcal {R}_0^{(i)}=\frac{a_i^2b_ic_i V_i}{\gamma _i\mu _i H_i^*}. \end{aligned}$$

Since the matrix $\mathscr {A}{\mathscr {D}}^{-1}\mathscr {B}{\mathscr {C}}^{-1}$ has essentially the same structure as that of the SIS patch model, we have the following result on the monotonicity of $\mathcal {R}_0$ in terms of host dispersal rate (Allen et al. 2007; Gao 2019; Gao and Dong 2020, and Chen et al. 2020).

### Proposition 2.1

For model (2.2), the basic reproduction number $\mathcal {R}_0(\varepsilon )$ is strictly decreasing and strictly convex in dispersal rate $\varepsilon \in [0,\infty )$ if $\mathcal {R}_0^{(i)}$ is nonconstant in $i\in \Omega$ and constant otherwise. Moreover, the inequality

$$\min _{i\in \Omega }\mathcal {R}_0^{(i)}<\mathcal {R}_0(\infty ) =\frac{\sum \nolimits _{i\in \Omega }\mathcal {R}_0^{(i)}\gamma _i\alpha _i}{\sum \nolimits _{i\in \Omega }\gamma _i\alpha _i}<\mathcal {R}_0(\varepsilon )<\mathcal {R}_0(0)=\max _{i\in \Omega }\mathcal {R}_0^{(i)},\quad \forall \varepsilon >0$$

holds if $\mathcal {R}_0^{(i)}$ is nonconstant in $i\in \Omega$. In addition, if $\mathcal {R}_0(0)>1$, then

- if $\mathcal {R}_0(\infty )<1$, then there is a threshold value $\varepsilon ^*\in (0,\infty )$ such that $\mathcal {R}_0(\varepsilon )>1$ for $\varepsilon <\varepsilon ^*$, $\mathcal {R}_0(\varepsilon )=1$ for $\varepsilon =\varepsilon ^*$, and $\mathcal {R}_0(\varepsilon )<1$ for $\varepsilon >\varepsilon ^*$;
- if $\mathcal {R}_0(\infty )\ge 1$, then $\mathcal {R}_0(\varepsilon )>1$ for all $\varepsilon \ge 0$.

In a homogeneous environment where hosts and vectors are epidemiologically and demographically identical across all patches, namely

$$\begin{aligned} a_i=a,\quad b_i=b,\quad c_i=c,\quad \gamma _i=\gamma ,\quad \mu _i=\mu ,\qquad i\in \Omega , \end{aligned}$$

the following result states that a nonhomogeneous mixing of hosts and vectors induced by host migration increases disease persistence.

### Proposition 2.2

(Corollary 1 in Gao et al. (2019)) Let $\mathcal {R}_0(n)$ be the basic reproduction number of the n-patch model (2.2) in terms of *n*, the number of patches. In a homogeneous environment, we have

$$\mathcal {R}_0(n)\ge \mathcal {R}_0(1)=\frac{a^2bc}{\gamma \mu }\sum _{i\in \Omega }V_i\Bigg / \sum _{i\in \Omega }H_i$$

with equality if and only if $(V_1,\dots ,V_n)^{\mathop {{\textrm{T}}}}$ is a right eigenvector of *L* corresponding to the zero eigenvalue, i.e., the distributions of hosts and vectors are uniform.

By the theory of monotone dynamical systems (Smith 1995; Zhao 2017), the disease dynamics of system (2.2) are fully determined by its basic reproduction number.

### Theorem 2.3

(Theorem 3.1 in Auger et al. (2008) and Theorem 2 in Cosner et al. (2009)) For model (2.2), if $\mathcal {R}_0\le 1$ then the disease-free equilibrium $(\varvec{0},\varvec{0})$ is globally asymptotically stable (GAS) among nonnegative solutions whereas if $\mathcal {R}_0>1$ then there is a unique positive equilibrium $E^*=(\varvec{h}^*,\varvec{v}^*)=(h_1^*,\dots ,h_n^*,v_1^*,\dots ,v_n^*)$ which is GAS among positive solutions.

## General results

For the *n*-patch model (2.2), we study the impact of host dispersal on both local and global host disease prevalences at the endemic equilibrium $E^*$. Specifically, these are defined as:

$$\begin{aligned} \mathcal {P}^{(i)}(\varepsilon )=\frac{h_i^*(\varepsilon )}{H_i^*}\quad \text{ and } \quad \mathcal {P}_n(\varepsilon )=\frac{T_n(\varepsilon )}{H}, \end{aligned}$$

where $T_n(\varepsilon )=\sum _{i\in \Omega } h_i^*(\varepsilon )$ and $H=\sum _{i\in \Omega }H_i^*$ are the total number of infected hosts and the total host population across all patches, respectively. The reason that the prevalence is evaluated at the endemic equilibrium $E^*$ is that all positive solutions of model (2.2) eventually converge to $E^*$ when $\mathcal {R}_0>1$. We start with a key lemma that reduces the number of equilibrium equations from 2*n* to *n*.

### Lemma 3.1

Suppose that (A1)–(A3) hold and $\mathcal {R}_0(\varepsilon )>1$ for some $\varepsilon >0$. Then $(h_1^*,\dots ,h_n^*,$ $v_1^*,\dots ,v_n^*)$ is the endemic equilibrium of system (2.2) if and only if

3.1 $$\begin{aligned} \begin{aligned}&(h_1^*,\dots ,h_n^*,v_1^*,\dots ,v_n^*)=\left( H I_1^*,\dots ,H I_n^*,\frac{\sigma _1I_1^*}{\sigma _1 I_1^*+\alpha _1}V_1,\dots ,\frac{\sigma _nI_n^*}{\sigma _n I_n^*+\alpha _n}V_n\right) , \end{aligned} \end{aligned}$$

where $\varvec{I}^*=(I_1^*,\dots ,I_n^*){=(h_1^*/H,\dots ,h_n^*/H)}$ is the unique positive solution to

3.2 $$\begin{aligned} \begin{aligned} \beta _i\left( 1-\frac{\sigma _i+1}{\sigma _i I_i+\alpha _i}I_i\right) I_i -\gamma _i I_i+\varepsilon \sum _{j\in \Omega }L_{ij}I_j=0,\quad i\in \Omega \end{aligned} \end{aligned}$$

in the region $\Gamma =\{(I_1,\cdots ,I_n)\in \mathbb {R}_+^n\ |\ 0\le I_i\le \alpha _i,\ i\in \Omega \}$ and

$$\beta _i=\frac{a_i^2b_ic_iV_i}{H\alpha _i\mu _i}=\frac{a_ib_iV_i}{H\alpha _i}\sigma _i =\mathcal {R}_0^{(i)}\gamma _i\quad \text{ and }\quad \sigma _i=\frac{a_ic_i}{\mu _i}.$$

### Proof

The endemic equilibrium of system (2.2) satisfies

3.3a $$\begin{aligned}&a_ib_i\frac{v_i^*}{H_i^*}(H_i^*-h_i^*)-\gamma _i h_i^*+\varepsilon \sum \limits _{j\in \Omega }L_{ij}h_j^*=0,\quad i\in \Omega , \end{aligned}$$

3.3b $$\begin{aligned}&a_ic_i\frac{h_i^*}{H_i^*}(V_i-v_i^*)-\mu _i v_i^*=0,\quad i\in \Omega . \end{aligned}$$

Denote $I_i^*=h_i^*/H$ for $i\in \Omega$. Solving $v_i^*$ from (3.3b) gives

3.4 $$\begin{aligned} \begin{aligned} v_i^*=\frac{a_ic_i\frac{h_i^*}{H_i^*}V_i}{a_ic_i\frac{h_i^*}{H_i^*}+\mu _i} =\frac{a_ic_iV_i}{a_ic_ih_i^*+\mu _iH_i^*}h_i^*=\frac{\frac{a_ic_i}{\mu _i}V_i}{\frac{a_ic_i}{\mu _i}\cdot \frac{h_i^*}{H}+\frac{H_i^*}{H}}\cdot \frac{h_i^*}{H} =\frac{\sigma _iV_i}{\sigma _i I_i^*+\alpha _i}I_i^* \end{aligned} \end{aligned}$$

and substituting it into the infection term of (3.3a) yields

$$\begin{aligned} \begin{aligned}&a_ib_i\frac{v_i^*}{H_i^*}(H_i^*-h_i^*) = a_ib_i\frac{\sigma _iV_i}{\sigma _i I_i^*+\alpha _i}I_i^*\cdot \frac{H_i^*-h_i^*}{H_i^*}\\=&\frac{a_ib_i\sigma _iV_i}{\sigma _i I_i^*+\alpha _i}\left( 1-\frac{I_i^*}{\alpha _i}\right) I_i^* =\frac{a_ib_i\sigma _iV_i}{\alpha _i}\cdot \frac{\alpha _i-I_i^*}{\sigma _i I_i^*+\alpha _i}I_i^*\\=&\frac{a_ib_i\sigma _iV_i}{\alpha _i}\left( 1-\frac{\sigma _i+1}{\sigma _i I_i^*+\alpha _i}I_i^*\right) I_i^*=\beta _i H\left( 1-\frac{\sigma _i+1}{\sigma _i I_i^*+\alpha _i}I_i^*\right) I_i^*. \end{aligned} \end{aligned}$$

Then dividing both sides of (3.3a) by *H* gives (3.2). The existence and uniqueness of positive solution to (3.2) are guaranteed by Theorem 2.3, or Theorem 3.1 in Gao and Ruan (2011). $\square$

### Remark 3.2

The system associated with (3.2), given by

3.5 $$\begin{aligned} \begin{aligned} \frac{dI_i}{dt}=\beta _i\left( 1-\frac{\sigma _i+1}{\sigma _i I_i+\alpha _i}I_i\right) I_i -\gamma _i I_i+\varepsilon \sum _{j\in \Omega }L_{ij}I_j,\quad i\in \Omega \end{aligned} \end{aligned}$$

can be viewed as the limiting system of an SIS patch model with equal migration rates but unequal contact rates between susceptible and infected populations (Brauer 2011) or unanimous infection-dependent behavior change (Gao and Ruan 2011). In fact, the infection term in (3.5) can be rewritten as

$$\beta _i\frac{\alpha _i-I_i}{\sigma _i I_i+\alpha _i}I_i=\frac{\beta _i}{\sigma _i+1}\cdot \frac{(\sigma _i+1)I_i}{(\sigma _i+1)I_i+(\alpha _i-I_i)}(\alpha _i-I_i),$$

where the ratio of contact rates of infected to susceptible people is $\sigma _i+1$, or rewritten as

$$\begin{aligned} \frac{a_ib_i\sigma _iV_i}{\sigma _i I_i+\alpha _i}\cdot \frac{I_i}{\alpha _i}(\alpha _i-I_i), \end{aligned}$$

where the transmission rate $\frac{a_ib_i\sigma _iV_i}{\sigma _i I_i+\alpha _i}$ is decreasing in $I_i$. Notably, if $\sigma _i=0$ for $i\in \Omega$, then the model (3.5) is exactly the SIS patch model with standard incidence and equal migration rates. Moreover, the multi- and single-patch reproduction numbers of both models (2.2) and (3.5) are respectively

$$\mathcal {R}_0=\rho (FV^{-1})\quad \text{ and } \quad \mathcal {R}_0^{(i)}=\frac{\beta _i}{\gamma _i}$$

with $F=\mathop {{\textrm{diag}}}(\beta _i)$ and $V=\mathop {{\textrm{diag}}}(\gamma _i)-\varepsilon L$, which are the same as the SIS patch model with standard incidence (see e.g., Allen et al. 2007).

Based on Lemma 3.1, the local and global host and vector disease prevalences can be simply expressed in terms of the positive solution of (3.2). By applying the comparison principle to the irreducible cooperative system (3.5), it follows that all these disease prevalences are strictly increasing in $a_i, b_i, c_i$ and $V_i$, but strictly decreasing in $\gamma _i, \mu _i$ and *H*, which are consistent with the disease persistence.

### Corollary 3.3

For model (2.2), if $\mathcal {R}_0(\varepsilon )>1$ for some $\varepsilon >0$, then the local and global disease prevalences for hosts and vectors are

$$\begin{aligned} \mathcal {P}^{(i)}(\varepsilon )=\frac{I_i^*(\varepsilon )}{\alpha _i}\quad \text{ and } \quad \mathcal {P}_n(\varepsilon )=\sum _{i\in \Omega } I_i^*(\varepsilon ), \end{aligned}$$

and

$$\mathscr {P}^{(i)}(\varepsilon )=\frac{\sigma _iI_i^*(\varepsilon )}{\sigma _i I_i^*(\varepsilon )+\alpha _i}\quad \text{ and } \quad \mathscr {P}_n(\varepsilon )=\sum _{i\in \Omega } \frac{\sigma _iI_i^*(\varepsilon )}{\sigma _i I_i^*(\varepsilon )+\alpha _i}V_i\Bigg / \sum _{i\in \Omega }V_i,$$

respectively, where $(I_1^*,\dots ,I_n^*)$ is the unique positive solution of (3.2).

### Remark 3.4

In a disconnected patch *i*, if $\mathcal {R}_0^{(i)}>1$ then

$$\begin{aligned} \begin{aligned} (h_i^*,v_i^*)&=\left( \frac{a_i^2b_ic_iV_i-\gamma _i\mu _iH_i^*}{a_i^2b_ic_iV_i+a_ic_i\gamma _iH_i^*}H_i^*, \frac{a_i^2b_ic_iV_i-\gamma _i\mu _iH_i^*}{a_i^2b_ic_i+a_ib_i\mu _i}\right) \\&=\left( \frac{\mathcal {R}_0^{(i)}-1}{\mathcal {R}_0^{(i)}+\sigma _i}H_i^*, \frac{\mathcal {R}_0^{(i)}-1}{\mathcal {R}_0^{(i)}(1+1/\sigma _i)}V_i\right) . \end{aligned} \end{aligned}$$

Inspired by the relationship between the basic reproduction number and disease prevalence in the single-patch SIS model (Gao 2020; Gao and Lou 2021), the host and vector disease prevalences of patch *i* in disconnection are written as

3.6 $$\begin{aligned} \begin{aligned} \mathcal {P}^{(i)}(0)=\left( 1-\frac{1}{\mathcal {R}_h^{(i)}}\right) ^+ \le \left( 1-\frac{1}{\mathcal {R}_0^{(i)}}\right) ^+, \end{aligned} \end{aligned}$$

and

$$\begin{aligned} \begin{aligned} \mathscr {P}^{(i)}(0)=\left( 1-\frac{1}{\mathcal {R}_v^{(i)}}\right) ^+ \le \left( 1-\frac{1}{\mathcal {R}_0^{(i)}}\right) ^+, \end{aligned} \end{aligned}$$

respectively, with equality if and only if $\mathcal {R}_0^{(i)}\le 1$, where $\zeta ^+=\max \{\zeta ,0\}$ and the host and vector patch reproduction numbers are respectively defined as

$$\mathcal {R}_h^{(i)}:=\frac{\beta _i+\sigma _i\gamma _i}{(1+\sigma _i)\gamma _i} =\frac{\mathcal {R}_0^{(i)}+\sigma _i}{1+\sigma _i}\quad \text{ and }\quad \mathcal {R}_v^{(i)}:=\frac{(1+\sigma _i)\beta _i}{\beta _i+\sigma _i\gamma _i} =\frac{(1+\sigma _i)\mathcal {R}_0^{(i)}}{\mathcal {R}_0^{(i)}+\sigma _i}$$

satisfying $\mathcal {R}_h^{(i)}\mathcal {R}_v^{(i)}=\mathcal {R}_0^{(i)}$. The units of parameters $\beta _i$ and $\sigma _i$ are per unit time and dimensionless, respectively. The three quantities $\mathcal {R}_0^{(i)}-1$, $\mathcal {R}_h^{(i)}-1$ and $\mathcal {R}_v^{(i)}-1$ have the same sign and $\mathop {{\textrm{sgn}}}(\mathcal {R}_0^{(i)}-\mathcal {R}_h^{(i)})=\mathop {{\textrm{sgn}}}(\mathcal {R}_0^{(i)}-\mathcal {R}_v^{(i)}) =\mathop {{\textrm{sgn}}}(\mathcal {R}_0^{(i)}-1)$. A patch may have a larger $\mathcal {R}_0^{(i)}$ yet a smaller $\mathcal {R}_h^{(i)}$, underscoring the necessity of measuring disease transmission using the host reproduction number in addition to the basic reproduction number.

### Remark 3.5

Note that if $\mathcal {P}^{(i)}(0)=\mathcal {P}^{(1)}(0)>0$, i.e., $\mathcal {R}_h^{(i)}=\mathcal {R}_h^{(1)}>1$, for all $i\in \Omega$, then the unique positive solution to equations (3.2) is given by

$$(I_1^*(\varepsilon ),\dots ,I_n^*(\varepsilon ))= \left( 1-\frac{1}{\mathcal {R}_h^{(1)}}\right) (\alpha _1,\dots ,\alpha _n)$$

and hence

$$\mathcal {P}^{(1)}(\varepsilon )=\cdots =\mathcal {P}^{(n)}(\varepsilon ) =\mathcal {P}_n(\varepsilon )=\mathcal {P}^{(1)}(0)=1-1/\mathcal {R}_h^{(1)},\quad \forall \varepsilon \ge 0.$$

Thus, the prevalences across patches are uniform. In other words, the distribution of infected hosts is proportional to the availability of hosts, which is analogous to the ideal free distribution (IFD) in ecology (Fretwell and Lucas 1969). For model (2.2), the basic reproduction number $\mathcal {R}_0(\varepsilon )$ is constant if and only if $\mathcal {R}_0^{(i)}$ is constant in $i\in \Omega$. Thus, the conditions for $\mathcal {R}_0(\varepsilon )$ and $\mathcal {P}_n(\varepsilon )$ to be constants are different. It is possible that $\mathcal {R}_0(\varepsilon )$ is constant while $\mathcal {P}_n(\varepsilon )$ varies. For example, consider a two-patch environment with $\beta _1=1,\gamma _1=1/2,\sigma _1=1/2,\beta _2=2,\gamma _2=1$, and $\sigma _2=1$, then $\mathcal {R}_0^{(1)}=\mathcal {R}_0^{(2)}=2$ but $\mathcal {R}_h^{(1)}=5/3\ne \mathcal {R}_h^{(2)}=3/2$. However, these two conditions align in the case of the SIS patch model with standard incidence (Allen et al. 2007).

To exclude the IFD case, we make an additional assumption hereafter:

(A4) the host patch reproduction number $\mathcal {R}_h^{(i)}$ is nonconstant in $i\in \Omega$.

We next estimate the local disease prevalence of any connected patch by the minimum and maximum disease prevalences of all disconnected patches, and establish a weak order-preserving property that correlates local disease prevalences with and without dispersal. The proofs are omitted since they are quite similar to those of Proposition 3.8 and Theorem 3.9 in Gao (2020), and Proposition 2.2 and Theorem 2.3 in Gao and Lou (2021).

### Proposition 3.6

For model (2.2), if $\mathcal {R}_0(\varepsilon )>1$ for some $\varepsilon >0$, then the local host disease prevalence at the endemic equilibrium $E^*$ satisfies

$$\begin{aligned} \min _{j\in \Omega } \mathcal {P}^{(j)}(0)<\mathcal {P}^{(i)}(\varepsilon )<\max _{j\in \Omega } \mathcal {P}^{(j)}(0),\quad i\in \Omega . \end{aligned}$$

Furthermore, the same estimate holds for the global host disease prevalence $\mathcal {P}_n(\varepsilon )$.

### Proposition 3.7

For model (2.2), suppose $\mathcal {P}^{(1)}(0)$ ≥ ⋯ ≥ $\mathcal {P}^{(n)}(0)$, i.e., $\mathcal {R}_h^{(1)}$ ≥ ⋯ ≥ $\mathcal {R}_h^{(n)}$, and $\mathcal {R}_0(\varepsilon )>1$ for some $\varepsilon >0$, then the local host disease prevalence at the endemic equilibrium $E^*$ satisfies

$$\min _{i\in \Omega }\mathcal {P}^{(i)}(\varepsilon )<\mathcal {P}^{(1)}(\varepsilon )<\mathcal {P}^{(1)}(0)\ \ \text{ and } \ \ \mathcal {P}^{(n)}(0)<\mathcal {P}^{(n)}(\varepsilon )<\max _{i\in \Omega }\mathcal {P}^{(i)}(\varepsilon ).$$

In particular, for the two-patch case, we always have $\mathcal {P}$^(1)^(0) > $\mathcal {P}$^(1)^(*ε*) > $\mathcal {P}$^(2)^(*ε*) > $\mathcal {P}$^(2)^(0).

Epidemiologically, these findings imply that dispersal has a diminishing/amplifying effect on the prevalence of the patch exhibiting the highest/lowest prevalence during disconnection. Nevertheless, the patch with the highest/lowest prevalence during disconnection cannot transit to the lowest/highest prevalence patch during connection. In what follows, we will focus on the global host disease prevalence, beginning with the limiting case whose proof is provided in “Appendix A”.

### Theorem 3.8

For model (2.2), the global host disease prevalence at $E^*$ satisfies

$$\mathcal {P}_n(0)=\sum _{i\in \Omega }I_i^*(0)=\sum _{i\in \Omega } \mathcal {P}^{(i)}(0)\alpha _i=\sum _{i\in \Omega }\left( 1-\frac{1}{\mathcal {R}_h^{(i)}}\right) ^+\alpha _i$$

and

$$\mathcal {P}_n(\infty )=\sum _{i\in \Omega }I_i^*(\infty )=\sum _{i\in \Omega } \mathcal {P}^{(i)}(\infty )\alpha _i=\tau _n,$$

where

$$\begin{aligned} \begin{aligned}&\mathcal {P}^{(i)}(0):=\lim _{\varepsilon \rightarrow 0+}\mathcal {P}^{(i)}(\varepsilon )=\left( 1-\frac{1}{\mathcal {R}_h^{(i)}}\right) ^+,\quad i\in \Omega , \\&\mathcal {P}^{(i)}(\infty ):=\lim _{\varepsilon \rightarrow \infty }\mathcal {P}^{(i)}(\varepsilon )=\tau _n,\quad i\in \Omega , \end{aligned} \end{aligned}$$

and $\tau _n\in (0,1-1/\mathcal {R}_0(\infty ))$ is the unique positive solution to

3.7 $$\begin{aligned} \begin{aligned}&\sum _{i\in \Omega }\left( \beta _i\frac{1-\tau _n}{\sigma _i\tau _n+1}-\gamma _i\right) \alpha _i=0 \end{aligned} \end{aligned}$$

if $\mathcal {R}_0(\infty )>1$, and $\tau _n=0$ if $\mathcal {R}_0(\infty )\le 1$.

### Remark 3.9

Under infinite dispersal, the local host disease prevalence across patches tends to the same level, $\tau _n$, but the local vector disease prevalence varies across patches. In fact, it follows from (3.4) that the vector disease prevalence of patch *i* is

$$\frac{v_i^*(\varepsilon )}{V_i}=\frac{\sigma _iI_i^*(\varepsilon )}{\sigma _iI_i^*(\varepsilon )+\alpha _i}=\frac{\sigma _i\mathcal {P}^{(i)}(\varepsilon )}{\sigma _i\mathcal {P}^{(i)}(\varepsilon )+1}\rightarrow \frac{\sigma _i\tau _n}{\sigma _i\tau _n+1},\quad \text{ as } \ \varepsilon \rightarrow \infty .$$

This also gives the global vector disease prevalence under infinite dispersal

$$\lim _{\varepsilon \rightarrow \infty }\frac{\sum _{i\in \Omega }v_i^*(\varepsilon )}{\sum _{i\in \Omega }V_i} =\sum _{i\in \Omega }\frac{\sigma _i\tau _n}{\sigma _i\tau _n+1}V_i\Bigg /\sum _{i\in \Omega }V_i.$$

Notably, if $\sigma _i=\sigma$ for all $i\in \Omega$, then the global/local host and vector disease prevalences are

$$\tau _n=\frac{\mathcal {R}_0(\infty )-1}{\mathcal {R}_0(\infty )+\sigma }\quad {\text{and}}\quad \frac{\sigma \tau _n}{\sigma \tau _n+1}=\frac{\sigma (\mathcal {R}_0(\infty )-1)}{(\sigma +1)\mathcal {R}_0(\infty )} \quad \text{with}\ \ \mathcal {R}_0(\infty )=\frac{\sum _{i\in \Omega }\beta _i\alpha _i}{\sum _{i\in \Omega }\gamma _i\alpha _i},$$

respectively. An interesting observation is that the susceptible ratio (proportion of people staying susceptible) for the single-patch SIS endemic model with standard incidence is $1/\mathcal {R}_0$, which remains true for the multi-patch case with infinite dispersal (Gao and Lou 2021). However, for the single-patch Ross–Macdonald model, $1/\mathcal {R}_0$ is not the susceptible ratio of hosts, but the geometric mean of the susceptible ratios of both hosts and vectors when $\mathcal {R}_0$ takes the square root form, see Auger et al. (2008), Cosner et al. (2009). Unfortunately, this relationship is generally invalid for the multi-patch case with infinite dispersal when $\sigma _i=a_ic_i/\mu _i$ is nonconstant in $i\in \Omega$.

Under certain conditions, we find that massive host dispersal can result in either more or fewer host infections compared to very limited dispersal. The detailed derivation is given in “Appendix B”. Recall that $\mathcal {R}_0(\varepsilon )$ is monotone decreasing. Therefore, an inconsistency between disease persistence and disease prevalence in response to fast dispersal may arise.

### Proposition 3.10

For model (2.2), suppose $\mathcal {R}_0^{(i)}>1$ and $\sigma _i=\sigma$ for all $i\in \Omega$, then the following statements hold for $\mathcal {P}_n(\varepsilon )$:

- if $\beta _i=\beta$ for all $i\in \Omega$, then $\mathcal {P}_n(\infty )\le \mathcal {P}_n(0)$ with equality if and only if $\gamma _i=\gamma$ for all $i\in \Omega$;
- if $\gamma _i=\gamma$ for all $i\in \Omega$, then $\mathcal {P}_n(\infty )\ge \mathcal {P}_n(0)$ with equality if and only if $\beta _i=\beta$ for all $i\in \Omega$;
- if $\beta _i-\gamma _i=c$ for all $i\in \Omega$, then $\mathcal {P}_n(\infty )\le \mathcal {P}_n(0)$ with equality if and only if $\beta _i=\beta$ for all $i\in \Omega$.

In reality, it is very likely that different patches have close recovery rates but different transmission rates, i.e., case (b), which implies that large dispersal causes more host infections. The condition $\beta _i-\gamma _i=c$ means that the spectral bound $s(F-V)$, related to the initial growth rate of new host infections, is constant in $\varepsilon$ (Gao and Dong 2020). Next we compute the right derivative of the global disease prevalence at zero dispersal, denoted by $\mathcal {P}_n'(0+)$, which can tell us when small dispersal causes more or less host infections than no dispersal.

### Proposition 3.11

For model (2.2), if $\mathcal {R}_0^{(i)}\ne 1$, i.e., $\mathcal {R}_h^{(i)}\ne 1$, for all $i\in \Omega$, then

$$\begin{aligned} \mathcal {P}_n'(0+)=-\sum _{i\in \Omega }\left( \frac{1}{\chi _i}\sum _{j\in \Omega }L_{ij}I_j^*(0)\right) , \end{aligned}$$

where $$I_j^*(0)=\mathcal {P}^{(j)}(0)\alpha _j=(1-1/\mathcal {R}_h^{(j)})^+\alpha _j$$ and

$$\begin{aligned} \chi _i={\left\{ \begin{array}{ll} \beta _i-\gamma _i, & \text{ if } \ \mathcal {R}_0^{(i)}=\beta _i/\gamma _i<1, \\ -\frac{\beta _i-\gamma _i}{\beta _i}\cdot \frac{\beta _i+\sigma _i\gamma _i}{1+\sigma _i}=-(\beta _i-\gamma _i)\frac{\mathcal {R}_h^{(i)}}{\mathcal {R}_0^{(i)}}, & \text{ if } \ \mathcal {R}_0^{(i)}=\beta _i/\gamma _i>1. \end{array}\right. } \end{aligned}$$

### Proof

Suppose $\mathcal {R}_0(0)=\max _{i\in \Omega }\mathcal {R}_0^{(i)}>1$. Otherwise, $\mathcal {R}_0(\varepsilon )\le \mathcal {R}_0(0)\le 1$ for $\varepsilon \ge 0$ and the disease-free equilibrium is globally asymptotically stable which means $\mathcal {P}_n(\varepsilon )=0$ and hence the conclusion $\mathcal {P}_n'(0+)=0$ holds. The continuity of $\mathcal {R}_0$ in $\varepsilon$ implies that $\mathcal {R}_0(\varepsilon )>1$ for small enough $\varepsilon$. By Lemma 3.1, there is a unique positive solution $(I_1^*,\dots ,I_n^*)$ to (3.2), or equivalently,

3.8 $$\begin{aligned} \begin{aligned} M_n(I_1^*,\dots ,I_n^*)^{\mathop {{\textrm{T}}}}=\varvec{0}, \end{aligned} \end{aligned}$$

where

$$M_n=F-V-\mathop {{\textrm{diag}}}\left( \beta _i\frac{\sigma _i+1}{\sigma _i I_i^*+\alpha _i}I_i^*\right) .$$

Differentiating both sides of (3.8) with respect to $\varepsilon$ yields

$$\begin{aligned} \begin{aligned} \tilde{M}_n\left( \frac{dI_1^*}{d\varepsilon },\dots ,\frac{dI_n^*}{d\varepsilon }\right) ^{\mathop {{\textrm{T}}}} =-\left( \sum _{j\in \Omega }L_{1j}I_j^*,\dots ,\sum _{j\in \Omega }L_{nj}I_j^*\right) ^{\mathop {{\textrm{T}}}}, \end{aligned} \end{aligned}$$

where

$$\tilde{M}_n=M_n-\mathop {{\textrm{diag}}}\left( \beta _i\frac{\sigma _i+1}{(\sigma _i I_i^*+\alpha _i)^2}\alpha _iI_i^*\right) .$$

Since $M_n$ is essentially nonnegative and irreducible, it follows from the Perron–Frobenius theorem that $s(M_n)=0$ and hence $s(\tilde{M}_n)<s(M_n)=0$ and $\mathop {{\textrm{sgn}}}(|\tilde{M}_n|)=(-1)^n\ne 0$. Thus, $\tilde{M}_n$ is invertible and its inverse is strictly negative (Smith 1995). Therefore,

3.9 $$\begin{aligned} \begin{aligned} \left( \frac{dI_1^*}{d\varepsilon },\dots ,\frac{dI_n^*}{d\varepsilon }\right) ^{\mathop {{\textrm{T}}}} =-\tilde{M}_n^{-1}\left( \sum _{j\in \Omega }L_{1j}I_j^*,\dots ,\sum _{j\in \Omega }L_{nj}I_j^*\right) ^{\mathop {{\textrm{T}}}}. \end{aligned} \end{aligned}$$

We arrive at the final conclusion since a direct calculation gives

$$\begin{aligned} \lim _{\varepsilon \rightarrow 0+}\tilde{M}_n=\mathop {{\textrm{diag}}}(\chi _i), \end{aligned}$$

where

$$\begin{aligned} \begin{aligned} \chi _i&=\beta _i-\gamma _i-\beta _i\frac{\sigma _i+1}{\sigma _iI_i^*(0)+\alpha _i}I_i^*(0) -\beta _i\frac{\sigma _i+1}{(\sigma _iI_i^*(0)+\alpha _i)^2}\alpha _iI_i^*(0)\\&=-\beta _i\frac{\sigma _i+1}{(\sigma _iI_i^*(0)+\alpha _i)^2}\alpha _iI_i^*(0) =-\beta _i\frac{\sigma _i+1}{(\sigma _i\mathcal {P}^{(i)}(0)+1)^2}\mathcal {P}^{(i)}(0)\\&=-\beta _i\frac{\sigma _i+1}{(\sigma _i(1-1/\mathcal {R}_h^{(i)})+1)^2}\left( 1-\frac{1}{\mathcal {R}_h^{(i)}}\right) \\&=-\frac{(\beta _i-\gamma _i)(\beta _i+\sigma _i\gamma _i)}{(1+\sigma _i)\beta _i}<0 \end{aligned} \end{aligned}$$

if $\mathcal {R}_0^{(i)}>1$, and $\chi _i=\beta _i-\gamma _i-0-0=\beta _i-\gamma _i<0$ if $\mathcal {R}_0^{(i)}<1$. $\square$

### Remark 3.12

For two-patch case, if $\mathcal {R}_0(\varepsilon )>1$ for some $\varepsilon \ge 0$, then $\mathop {{\textrm{sgn}}}(\mathcal {P}_2'(0+))=\mathop {{\textrm{sgn}}}(\chi _2-\chi _1)\cdot \mathop {{\textrm{sgn}}}(\mathcal {R}_h^{(1)}-\mathcal {R}_h^{(2)})$. In fact, without loss of generality, we may assume that $\mathcal {R}_h^{(1)}>\mathcal {R}_h^{(2)}$. Then it follows from Proposition 3.7 that

$$\mathcal {P}^{(1)}(\varepsilon )=\frac{I_1^*(\varepsilon )}{\alpha _1}>\mathcal {P}^{(2)}(\varepsilon )=\frac{I_2^*(\varepsilon )}{\alpha _2}\ \Leftrightarrow \ L_{21}I_1^*(\varepsilon )-L_{12}I_2^*(\varepsilon )>0,\quad \forall \varepsilon \ge 0,$$

and hence

$$\mathop {{\textrm{sgn}}}(\mathcal {P}_2'(0+))=\mathop {{\textrm{sgn}}}\left( \frac{(\chi _2-\chi _1) (L_{21}I_1^*(0)-L_{12}I_2^*(0))}{\chi _1\chi _2}\right) =\mathop {{\textrm{sgn}}}(\chi _2-\chi _1).$$

## Two-patch case

In this section, we restrict our attention to the two-patch submodel

4.1 $$\begin{aligned} \begin{aligned}&\dfrac{dh_i}{dt}=a_ib_i\frac{v_i}{H_i^*}(H_i^*-h_i)-\gamma _i h_i+\varepsilon \sum \limits _{j=1}^2L_{ij}h_j,\quad 1\le i\le 2, \\&\dfrac{dv_i}{dt}=a_ic_i\frac{h_i}{H_i^*}(V_i-v_i)-\mu _i v_i,\quad 1\le i\le 2, \end{aligned} \end{aligned}$$

where $(H_1^*,H_2^*)=H(\alpha _1,\alpha _2)$ with $\alpha _1=\frac{L_{12}}{L_{12}+L_{21}}$ and $\alpha _2=\frac{L_{21}}{L_{12}+L_{21}}$. Recall that assumptions (A1)–(A4) are still required. In particular, these mean that $\mathcal {R}_h^{(1)}\ne \mathcal {R}_h^{(2)}$ and $L_{12}>0$ and $L_{21}>0$. The associated global host disease prevalence $\mathcal {P}_2(\varepsilon )$ equals zero if $\mathcal {R}_0(\varepsilon )\le 1$ and $I_1^*(\varepsilon )+I_2^*(\varepsilon )$ if $\mathcal {R}_0(\varepsilon )>1$ where $(I_1^*,I_2^*)\in (0,\alpha _1)\times (0,\alpha _2)$ satisfies

4.2 $$\begin{aligned} \begin{aligned}&\left( \beta _i-\gamma _i-\beta _i\frac{\sigma _i+1}{\sigma _i I_i^*+\alpha _i}I_i^*\right) I_i^* +\varepsilon \sum \limits _{j=1}^2L_{ij}I_j^*=0, \quad 1\le i\le 2. \end{aligned} \end{aligned}$$

For convenience, we introduce the following notations

$$g_i(I_i)=\beta _i-\gamma _i-\beta _i\frac{\sigma _i+1}{\sigma _i I_i+\alpha _i}I_i\ \text{ and } \ f_i(I_i)=g_i(I_i)-\frac{\beta _i\alpha _i(\sigma _i+1)}{(\sigma _iI_i+\alpha _i)^2}I_i,\quad i=1,2.$$

Using approaches similar to those developed by Gao and Lou (2021) for directly transmitted diseases, we will answer two basic questions: one concerns when dispersal leads to higher or lower global host disease prevalence than no dispersal, i.e., $\mathcal {P}_2(\varepsilon )$ versus $\mathcal {P}_2(0)$; the other involves how the global host disease prevalence changes with the host dispersal rate, i.e., the monotonicity of $\mathcal {P}_2(\varepsilon )$.

### Lemma 4.1

For model (4.1), if $0<\mathcal {P}_2(\varepsilon _0)\le \mathcal {P}_2(0)$ for some $\varepsilon _0>0$, then $\mathcal {P}_2'(\varepsilon _0)<0$. In particular, if $\mathcal {P}_2'(0+)<0$, then $\mathcal {P}_2'(\varepsilon )<0$ for all $\varepsilon \in (0,\infty )$ as $\mathcal {R}_0(\infty )\ge 1$ or $\varepsilon \in (0,\varepsilon ^*)$ as $\mathcal {R}_0(\infty )<1$ where

$$\mathcal {R}_0(\infty )=\frac{\beta _1L_{12}+\beta _2L_{21}}{\gamma _1L_{12}+\gamma _2L_{21}}, \quad \text{ and } \quad \varepsilon ^*=\frac{(\beta _1-\gamma _1)(\beta _2-\gamma _2)}{(\beta _1-\gamma _1)L_{12}+(\beta _2-\gamma _2)L_{21}}$$

is the unique positive root to $\mathcal {R}_0(\varepsilon )=1$ as $\mathcal {R}_0(\infty )<1$.

### Proof

It follows from (3.9) that

$$\begin{aligned} \begin{aligned} \left( \frac{dI_1^*}{d\varepsilon },\frac{dI_2^*}{d\varepsilon }\right) ^{\mathop {{\textrm{T}}}} =&-(L_{21}I_1^*-L_{12}I_2^*)\tilde{M}_2^{-1}\begin{pmatrix} -1 \\ 1 \end{pmatrix}, \end{aligned} \end{aligned}$$

where

$$\begin{aligned} \begin{aligned} \tilde{M}_2^{-1}&=\frac{1}{|\tilde{M}_2|}\begin{pmatrix} g_2(I_2^*)-\varepsilon L_{12}-\frac{\beta _2\alpha _2(\sigma _2+1)}{(\sigma _2I_2^*+\alpha _2)^2}I_2^* & -\varepsilon L_{12} \\ -\varepsilon L_{21} & g_1(I_1^*)-\varepsilon L_{21}-\frac{\beta _1\alpha _1(\sigma _1+1)}{(\sigma _1I_1^*+\alpha _1)^2}I_1^* \end{pmatrix} \\ &=\frac{-1}{|\tilde{M}_2|} \begin{pmatrix} \varepsilon L_{21}\frac{I_1^*}{I_2^*}+ \frac{\beta _2\alpha _2(\sigma _2+1)}{(\sigma _2I_2^*+\alpha _2)^2}I_2^* & \varepsilon L_{12} \\ \varepsilon L_{21} & \varepsilon L_{12}\frac{I_2^*}{I_1^*}+ \frac{\beta _1\alpha _1(\sigma _1+1)}{(\sigma _1I_1^*+\alpha _1)^2}I_1^* \end{pmatrix} \end{aligned} \end{aligned}$$

with $$|\tilde{M}_2|>0$$. Thus,

4.3 $$\begin{aligned} \nonumber&\frac{|\tilde{M}_2|}{L_{21}I_1^*-L_{12}I_2^*}\mathcal {P}_2'(\varepsilon )\nonumber \\=&\sum _{i=1}^2(-1)^i\left( g_i(I_i^*)-\frac{\beta _i\alpha _i(\sigma _i+1)}{(\sigma _iI_i^*+\alpha _i)^2}I_i^* \right) \nonumber \\=&\sum _{i=1}^2(-1)^i\left( \frac{-(\beta _i+\sigma _i\gamma _i)I_i^* +(\beta _i-\gamma _i)\alpha _i}{\sigma _iI_i^*+\alpha _i}- \frac{\beta _i\alpha _i(\sigma _i+1)}{(\sigma _iI_i^*+\alpha _i)^2}I_i^* \right) \nonumber \\=&\sum _{i=1}^2(-1)^i\left( -\frac{(\beta _i+\sigma _i\gamma _i)I_i^*}{\sigma _iI_i^*+\alpha _i}+\frac{\alpha _i}{\sigma _iI_i^*+\alpha _i} \left( \beta _i-\gamma _i-\frac{\beta _i(\sigma _i+1)}{\sigma _i I_i^*+\alpha _i}I_i^*\right) \right) \nonumber \\=&\left( \frac{(\beta _1+\sigma _1\gamma _1)I_1^*}{\sigma _1I_1^*+\alpha _1}-\frac{(\beta _2+\sigma _2\gamma _2)I_2^*}{\sigma _2I_2^*+\alpha _2}\right) \nonumber \\&-\frac{\alpha _1}{\sigma _1I_1^*+\alpha _1} g_1(I_1^*)+\frac{\alpha _2}{\sigma _2I_2^*+\alpha _2} g_2(I_2^*). \end{aligned}$$

We omit the rest of the proof which is similar to Lemma 4.1 in Gao and Lou (2021). $\square$

Biologically speaking, the lemma means that once the host infection size is no greater than that without dispersal, faster dispersal will keep reducing the number of infected hosts. We now proceed to compare the disease prevalence with and without dispersal. By using Lemma 4.1, it leaves only to consider the cases where $\mathcal {P}_2'(0+)=0$ and $\mathcal {P}_2'(0+)=\infty$. The interested reader is referred to Theorem 4.5 of Gao and Lou (2021) for a detailed argument.

### Theorem 4.2

Suppose $\mathcal {R}_0(0)=\max \limits _{1\le i\le 2}\mathcal {R}_0^{(i)}>1$ and $\mathcal {R}_h^{(1)}\ne \mathcal {R}_h^{(2)}$ for model (4.1). Then

- if $\mathcal {P}_2'(0+)\le 0$, then $\mathcal {P}_2(\varepsilon )<\mathcal {P}_2(0)$ for $\varepsilon \in (0,\infty )$;
- if $0<\mathcal {P}_2'(0+)\le \infty$ and $\mathcal {P}_2(\infty )<\mathcal {P}_2(0)$, then there is a unique positive $\hat{\varepsilon }$ such that $\mathcal {P}_2(\varepsilon )>\mathcal {P}_2(0)$ for $\varepsilon \in (0,\hat{\varepsilon })$, $\mathcal {P}_2(\varepsilon )=\mathcal {P}_2(0)$ for $\varepsilon =\hat{\varepsilon }$, and $\mathcal {P}_2(\varepsilon )<\mathcal {P}_2(0)$ for $\varepsilon \in (\hat{\varepsilon },\infty )$;
- if $\mathcal {P}_2(\infty )\ge \mathcal {P}_2(0)$, then $\mathcal {P}_2(\varepsilon )> \mathcal {P}_2(0)$ for $\varepsilon \in (0,\infty )$ (and $\mathcal {P}_2'(0+)>0$).

Before analyzing the monotonicity of the global host prevalence $\mathcal {P}_2(\varepsilon )$, we first examine the monotonicity of the local disease prevalence $\mathcal {P}^{(i)}(\varepsilon )=I_i^*(\varepsilon )/\alpha _i$. When dispersal is small, Proposition 3.6 indicates that increasing host dispersal reduces the disease prevalence of the patch with larger host patch reproduction number but increases that of the patch with smaller host patch reproduction number. We give a complete description below. By Corollary 3.3, this monotonicity result also applies to the local vector disease prevalence.

### Proposition 4.3

Suppose $\mathcal {R}_h^{(1)}>\max \{\mathcal {R}_h^{(2)},1\}$ for model (4.1). The following statements hold for the local host disease prevalence:

- if $\mathcal {R}_0(\infty )\ge 1$, then $\frac{d\mathcal {P}^{(1)}}{d\varepsilon }<0$ for $\varepsilon \in [0,\infty )$; if otherwise, then $\frac{d\mathcal {P}^{(1)}}{d\varepsilon }<0$ for $\varepsilon \in [0,\varepsilon ^*)$;
- if $f_1(I_1^*(\infty ))\le 0$, then $\frac{d\mathcal {P}^{(2)}}{d\varepsilon }>0$ for $\varepsilon \in [0,\infty )$; if otherwise, there exists some $\bar{\varepsilon }>0$ such that $\frac{d\mathcal {P}^{(2)}}{d\varepsilon }>0$ for $\varepsilon \in [0,\bar{\varepsilon })$, $\frac{d\mathcal {P}^{(2)}}{d\varepsilon }=0$ for $\varepsilon =\bar{\varepsilon }$, and $\frac{d\mathcal {P}^{(2)}}{d\varepsilon }<0$ for $\varepsilon \in (\bar{\varepsilon },\infty )$ when $\mathcal {R}_0(\infty )\ge 1$ or $\varepsilon \in (\bar{\varepsilon },\varepsilon ^*)$ when $\mathcal {R}_0(\infty )<1$.

### Proof

According to (4.3), we know

$$\begin{aligned} \mathop {{\textrm{sgn}}}((I_1^*)'(\varepsilon ))=\mathop {{\textrm{sgn}}}(f_2(I_2^*(\varepsilon )))\ \text{ and } \ \mathop {{\textrm{sgn}}}((I_2^*)'(\varepsilon ))=\mathop {{\textrm{sgn}}}(-f_1(I_1^*(\varepsilon ))). \end{aligned}$$

(a) It follows from $f_2(I_2^*)<g_2(I_2^*)<0$ that $\frac{dI_1^*}{d\varepsilon }=\alpha _1\frac{d\mathcal {P}^{(1)}}{d\varepsilon }<0$ on $[0,\infty )$ when $\mathcal {R}_0(\infty )\ge 1$ and on $[0,\varepsilon ^*)$ otherwise.

(b) Direct calculations give

4.4 $$\begin{aligned} f_i'(I_i)&=-\beta _i\alpha _i\frac{\sigma _i+1}{(\sigma _iI_i+\alpha _i)^2}\left( 1+\frac{\alpha _i-\sigma _iI_i}{\sigma _iI_i+\alpha _i}\right) \nonumber \\&=-2\beta _i\alpha _i^2\frac{\sigma _i+1}{(\sigma _iI_i+\alpha _i)^3}<0,\quad i=1,2. \end{aligned}$$

Combining (4.4) and part (a), we have

$$\frac{d}{d\varepsilon }(-f_1(I_1^*(\varepsilon )))=-f_1'(I_1^*(\varepsilon )) \frac{dI_1^*(\varepsilon )}{d\varepsilon }<0,$$

which means $-f_1(I_1^*(\varepsilon ))$ is strictly decreasing in $\varepsilon$. If $f_1(I_1^*(\infty ))\le 0$, i.e., $-f_1(I_1^*(\infty ))\ge 0$, then $-f_1(I_1^*(\varepsilon ))>0$ for all $\varepsilon >0$ and hence $\frac{dI_2^*}{d\varepsilon }=\alpha _2\frac{d\mathcal {P}^{(2)}}{d\varepsilon }>0$. $\square$

Next, we show that any nontrivial critical number of $\mathcal {P}_2(\varepsilon )$ corresponds to a local maximum. This means that the monotonicity of $\mathcal {P}_2(\varepsilon )$ follows one of the four patterns: strictly increasing, strictly decreasing, initially increasing and then decreasing, and constant.

### Lemma 4.4

For model (4.1), if $\mathcal {P}_2'(\varepsilon _0)=0$ and $\mathcal {R}_0(\varepsilon _0)>1$ for some $\varepsilon _0>0$, then $\mathcal {P}_2''(\varepsilon _0)<0$, i.e., every nontrivial critical number (the associated function value is nonzero) of $\mathcal {P}_2(\varepsilon )$ corresponds to a local maximum. Furthermore, $\mathcal {P}_2(\varepsilon )$ has at most one nontrivial critical number $\varepsilon _0\in \mathbb {R}_+$ and (if exists) $\mathcal {P}_2'(\varepsilon )<0$ for all $\varepsilon \in (\varepsilon _0,\infty )$ when $\mathcal {R}_0(\infty )\ge 1$ or $\varepsilon \in (\varepsilon _0,\varepsilon ^*)$ when $\mathcal {R}_0(\infty )<1$.

### Proof

Suppose $\mathcal {R}_0(\varepsilon )>1$. Differentiating the equations (4.2) in $\varepsilon$ yields

4.5 $$\begin{aligned} \begin{aligned}&f_i(I_i^*)\cdot (I_i^*)' +\varepsilon \sum \limits _{j=1}^2L_{ij}(I_j^*)'+\sum \limits _{j=1}^2L_{ij}I_j^*=0, \quad i=1,2. \end{aligned} \end{aligned}$$

Summing up (4.5) over $i\in \{1,2\}$ gives

4.6 $$\begin{aligned} \begin{aligned}&\sum _{i=1}^2f_i(I_i^*)\cdot (I_i^*)'=0. \end{aligned} \end{aligned}$$

Assume $\mathcal {P}_2'(\varepsilon _0)$ = $(I_1^*)'(\varepsilon _0)+(I_2^*)'(\varepsilon _0)$ = 0, then we claim that $(I_1^*)'(\varepsilon _0)=-(I_2^*)'(\varepsilon _0)\ne 0$. Suppose not, then $(I_1^*)'(\varepsilon _0)=(I_2^*)'(\varepsilon _0)=0$ and hence evaluating (4.5) at $\varepsilon =\varepsilon _0$ gives $L_{21}I_1^*(\varepsilon _0)-L_{12}I_2^*(\varepsilon _0)=0$, contradicting Proposition 3.7 or Remark 3.12. Thus, it follows from (4.6) that $f_1(I_1^*(\varepsilon _0))-f_2(I_2^*(\varepsilon _0))=0$ and hence

$$\begin{aligned} \begin{aligned}&\eta :=f_1(I_1^*(\varepsilon _0))=f_2(I_2^*(\varepsilon _0))<0. \end{aligned} \end{aligned}$$

The negativity of $\eta$ is due to

$$\eta (I_1^*(\varepsilon _0)+I_2^*(\varepsilon _0))=\sum _{i=1}^2 f_i(I_i^*(\varepsilon _0))I_i^*(\varepsilon _0) <\sum _{i=1}^2g_i(I_i^*(\varepsilon _0))I_i^*(\varepsilon _0) =0.$$

Differentiating again in (4.6) and applying (4.4) yield

4.7 $$\begin{aligned} \sum _{i=1}^2f_i(I_i^*)\cdot (I_i^*)''&=-\sum _{i=1}^2f_i'(I_i^*)\cdot ((I_i^*)')^2\nonumber \\&=\sum _{i=1}^2 2\beta _i\alpha _i^2\frac{\sigma _i+1}{(\sigma _iI_i^*+\alpha _i)^3}((I_i^*)')^2. \end{aligned}$$

Evaluating (4.7) at $\varepsilon =\varepsilon _0$ gives

$$\sum _{i=1}^2\eta (I_i^*)''(\varepsilon _0)=\eta \mathcal {P}_2''(\varepsilon _0) =\sum _{i=1}^2 2\beta _i\alpha _i^2\frac{\sigma _i+1}{(\sigma _iI_i^*(\varepsilon _0)+\alpha _i)^3}((I_i^*)'(\varepsilon _0))^2>0.$$

Hence $\mathcal {P}_2''(\varepsilon _0)<0$. By the second derivative test, $\mathcal {P}_2(\varepsilon )$ has a local maximum at $\varepsilon =\varepsilon _0$. $\square$

To distinguish the strictly increasing pattern from the initially increasing then decreasing pattern, we define a quantity to decide the monotonicity of $\mathcal {P}_2(\varepsilon )$ under large dispersal.

### Lemma 4.5

For model (4.1), if $\mathcal {R}_0(\infty )>1$ and

$$\mathbb {P}_2'(\infty ):=\mathop {{\textrm{sgn}}}(\mathcal {R}_h^{(1)}-\mathcal {R}_h^{(2)})\cdot \sum _{i=1}^2(-1)^i\left( \beta _i\frac{1-2\tau _2-\sigma _i\tau _2^2}{(\sigma _i\tau _2+1)^2}-\gamma _i \right) \ne 0,$$

then $\mathop {{\textrm{sgn}}}(\mathcal {P}_2'(\varepsilon ))=\mathop {{\textrm{sgn}}}(\mathbb {P}_2'(\infty ))$ for sufficiently large $\varepsilon$ where $\tau _2=\mathcal {P}_2(\infty )\in (0,1)$ is the unique positive solution to

$$\begin{aligned} \begin{aligned}&\sum _{i=1}^2\left( \beta _i\frac{1-\tau _2}{\sigma _i\tau _2+1}-\gamma _i\right) \alpha _i=0. \end{aligned} \end{aligned}$$

### Proof

Following (4.3), we have

4.8 $$\begin{aligned} \begin{aligned}&\mathcal {P}_2'(\varepsilon ) =\frac{L_{21}I_1^*-L_{12}I_2^*}{|\tilde{M}_2|}\times \sum _{i=1}^2(-1)^i\left( g_i(I_i^*)-\frac{\beta _i\alpha _i(\sigma _i+1)}{(\sigma _iI_i^*+\alpha _i)^2}I_i^* \right) . \end{aligned} \end{aligned}$$

Applying Remark 3.12, we know $\mathop {{\textrm{sgn}}}(L_{21}I_1^*-L_{12}I_2^*)=\mathop {{\textrm{sgn}}}(\mathcal {R}_h^{(1)}-\mathcal {R}_h^{(2)})$. Replacing $(I_1^*,I_2^*)$ by $(I_1^*(\infty ),I_2^*(\infty ))=\tau _2(\alpha _1,\alpha _2)$ in the second term of (4.8) yields

$$\begin{aligned}&\sum _{i=1}^2(-1)^{i}\left( \beta _i-\gamma _i-\frac{\beta _i(\sigma _i+1)}{\sigma _i \tau _2+1}\tau _2-\frac{\beta _i(\sigma _i+1)}{(\sigma _i\tau _2+1)^2}\tau _2 \right) \\=&\sum _{i=1}^2(-1)^i\left( \beta _i\frac{1-2\tau _2-\sigma _i\tau _2^2}{(\sigma _i\tau _2+1)^2}-\gamma _i \right) , \end{aligned}$$

where $\tau _2\in (0,1)$ is the limiting global disease prevalence satisfying

$$\begin{aligned} \begin{aligned}&\sum _{i=1}^2\left( \beta _i\frac{1-\tau _2}{\sigma _i\tau _2+1}-\gamma _i\right) \alpha _i=0, \end{aligned} \end{aligned}$$

and can be solved explicitly from the quadratic equation

$$\begin{aligned}&\sum _{\begin{array}{c} i,j=1\\ j\ne i \end{array}}^2(\beta _i+\gamma _i\sigma _i)\sigma _j\alpha _i\tau _2^2+ \sum _{\begin{array}{c} i,j=1\\ j\ne i \end{array}}^2(\beta _i(1-\sigma _j)+\gamma _i(\sigma _1+\sigma _2))\alpha _i\tau _2\\&\quad -\sum _{i=1}^2(\beta _i-\gamma _i)\alpha _i=0. \end{aligned}$$

Clearly, $\mathcal {P}_2'(\varepsilon )$ and $\mathbb {P}_2'(\infty )$ have the same sign when $\varepsilon$ is large enough. $\square$

To keep disease persistent at certain dispersal, we classify the monotonic pattern of the global host disease prevalence $\mathcal {P}_2(\varepsilon )$ under $\mathcal {R}_0(0)=\max \{\mathcal {R}_0^{(1)},\mathcal {R}_0^{(2)}\}>1$, or equivalently, $\max \{\mathcal {R}_h^{(1)},\mathcal {R}_h^{(2)}\}>1$.

### Theorem 4.6

For model (4.1), suppose $\mathcal {R}_0(0)>1$ and $\mathcal {R}_h^{(1)}\ne \mathcal {R}_h^{(2)}$ . Then

- if $\mathcal {P}_2'(0+)\le 0$, then $\mathcal {P}_2'(\varepsilon )<0$ for $\varepsilon \in (0,\infty )$ as $\mathcal {R}_0(\infty )\ge 1$, or $\mathcal {P}_2'(\varepsilon )<0$ for $\varepsilon \in (0,\varepsilon ^*)$ and $\mathcal {P}_2'(\varepsilon )=0$ for $\varepsilon \in (\varepsilon ^*,\infty )$ as $\mathcal {R}_0(\infty )<1$;
- if $0<\mathcal {P}_2'(0+)\le \infty$ and $\mathcal {R}_0(\infty )<1$, then there is a unique $\varepsilon _1\in (0,\varepsilon ^*)$ such that $\mathcal {P}_2'(\varepsilon )>0$ for $\varepsilon \in (0,\varepsilon _1)$, $\mathcal {P}_2'(\varepsilon )=0$ for $\varepsilon =\varepsilon _1$, $\mathcal {P}_2'(\varepsilon )<0$ for $\varepsilon \in (\varepsilon _1,\varepsilon ^*)$, and $\mathcal {P}_2'(\varepsilon )=0$ for $\varepsilon \in (\varepsilon ^*,\infty )$;
- if $0<\mathcal {P}_2'(0+)\le \infty$, $\mathcal {R}_0(\infty )\ge 1$ and $\mathbb {P}_2'(\infty )\ge 0$, then $\mathcal {P}_2'(\varepsilon )>0$ for $\varepsilon \in (0,\infty )$;
- if $0<\mathcal {P}_2'(0+)\le \infty$, $\mathcal {R}_0(\infty )\ge 1$ and $\mathbb {P}_2'(\infty )<0$, then there is a unique positive $\varepsilon _2$ such that $\mathcal {P}_2'(\varepsilon )>0$ for $\varepsilon \in (0,\varepsilon _2)$, $\mathcal {P}_2'(\varepsilon )=0$ for $\varepsilon =\varepsilon _2$, $\mathcal {P}_2'(\varepsilon )<0$ for $\varepsilon \in (\varepsilon _2,\infty )$.

### Proof

According to Theorem 4.2 and Lemmas 4.4 and 4.5, it only leaves to show $\mathcal {P}_2'(\varepsilon )>0$ for all $\varepsilon >0$ under $0<\mathcal {P}_2'(0+)\le \infty$, $\mathcal {R}_0(\infty )\ge 1$ and $\mathbb {P}_2'(\infty )=0$.

Without loss of generality, let $\mathcal {R}_h^{(1)}>\mathcal {R}_h^{(2)}$. It follows from (4.8) that

$$\begin{aligned} \mathop {{\textrm{sgn}}}(\mathcal {P}_2'(\varepsilon ))=\mathop {{\textrm{sgn}}}(\mathbb {P}_2'(\varepsilon )), \end{aligned}$$

where $\mathbb {P}_2'(\varepsilon ):=f_2(I_2^*(\varepsilon ))-f_1(I_1^*(\varepsilon ))$. Clearly, $\mathbb {P}_2'(\infty )=0$ implies $\mathcal {R}_0(\infty )>1$. Suppose not, it follows from $\mathop {{\textrm{sgn}}}(\mathcal {R}_h^{(i)}-1)=\mathop {{\textrm{sgn}}}(\mathcal {R}_0^{(i)}-1)$ that $\mathcal {R}_0(\infty )=1$ means $\mathcal {R}_0^{(1)}=\beta _1/\gamma _1>1>\mathcal {R}_0^{(2)}=\beta _2/\gamma _2$ and $I_1^*(\infty )=I_2^*(\infty )=0$. So,

$$\mathbb {P}_2'(\infty )=f_2(I_2^*(\infty ))-f_1(I_1^*(\infty ))= f_2(0)-f_1(0)=(\beta _2-\gamma _2)-(\beta _1-\gamma _1)<0,$$

a contradiction. The fact $\mathbb {P}_2'(\infty )=f_2(I_2^*(\infty ))-f_1(I_1^*(\infty ))=0$ implies that $f_1(I_1^*(\infty ))=f_2(I_2^*(\infty ))<g_2(I_2^*(\infty ))<0$. Applying Proposition 4.3, we know $(I_1^*)'(\varepsilon )<0$ and $(I_2^*)'(\varepsilon )>0$. Meanwhile, (4.4) implies $f_i'(I_i)<0$ for $i=1,2$. Thus,

$$\mathbb {P}_2''(\varepsilon )=f_2'(I_2^*(\varepsilon ))\cdot (I_2^*)'(\varepsilon ) -f_1'(I_1^*(\varepsilon ))\cdot (I_1^*)'(\varepsilon )<0,\quad \forall \varepsilon \in (0,\infty ).$$

Since $\mathbb {P}_2'(\varepsilon )$ is strictly decreasing and $\mathbb {P}_2'(\infty )=0$, then $\mathbb {P}_2'(\varepsilon )>0$ for $\varepsilon \in (0,\infty )$. $\square$

## Numerical simulations

In this section, mainly based on the two-patch submodel (4.1), we will numerically explore the dependence of the host disease prevalence on host dispersal rate, the inconsistency between host and vector disease prevalences, and the effects of nonhomogeneous mixing of hosts and vectors on disease prevalence and persistence. Recall that

$$\beta _i=\frac{a_ib_iV_i}{H\alpha _i}\sigma _i\quad \text{ and } \quad \sigma _i=\frac{a_ic_i}{\mu _i}.$$

Consequently, we can treat $\beta _i, \sigma _i$ and $\gamma _i$ in system (3.5) as mutually independent parameters. Parameter values are generally selected based on malaria epidemiology (Ruan et al. 2008), with days as the default time unit.

### Example 5.1

**Fig. 1:**
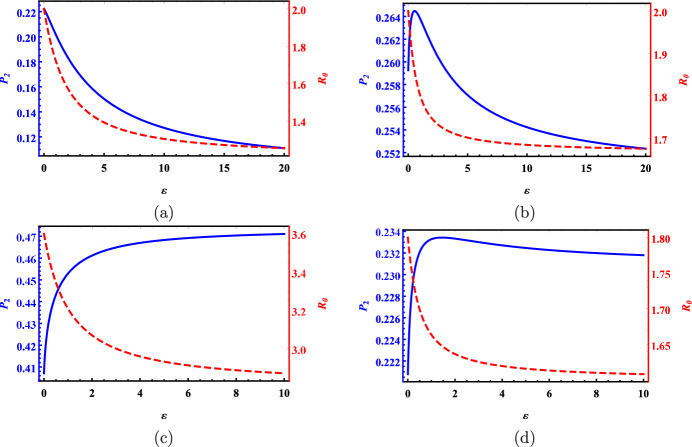
The global host disease prevalence $\mathcal {P}_2$ (blue solid line) and the basic reproduction number $\mathcal {R}_0$ (red dashed line) versus host dispersal rate $\varepsilon$ under four scenarios. The *x*-axis denotes $\varepsilon$ while the left and right *y*-axes are $\mathcal {P}_2$ and $\mathcal {R}_0$, respectively. Refer to Table 1 for parameter settings.

($\mathcal {P}_n(\varepsilon )$ and $\mathcal {P}^{(i)}(\varepsilon )$) For the two-patch submodel (4.1), we choose four parameter sets as summarized in Table 1 and plot the corresponding curves for global host disease prevalence $\mathcal {P}_2$ and the basic reproduction number $\mathcal {R}_0$ in terms of the host dispersal rate $\varepsilon$ in Fig. 1. In all four scenarios, patch one is the high-risk patch for hosts, characterized by a larger host patch reproduction number, i.e., $\mathcal {R}_h^{(1)}>\mathcal {R}_h^{(2)}$. The disease persists in both patches when dispersal occurs, as $\mathcal {R}_0(\varepsilon )>\mathcal {R}_0(\infty )>1$ for any $\varepsilon >0$. Furthermore, in line with Proposition 2.1, the red dashed line for $\mathcal {R}_0(\varepsilon )$ is monotonically decreasing and strictly convex in all scenarios.

**Table 1:** The parameter settings for all four scenarios in Fig. 1 with $\sigma _1=\sigma _2=1, L_{12}=0.02$, and $L_{21}=0.01.$

	$\beta _1$	$\gamma _1$	$\beta _2$	$\gamma _2$	$\mathcal {R}_h^{(1)}$	$\mathcal {R}_h^{(2)}$	$\mathcal {R}_0(\infty )$	$\mathcal {P}_2(0)$	$\mathcal {P}_2(\infty )$	$\mathcal {P}_2'(0+)$	$\mathbb {P}_2'(\infty )$
a	0.1	0.05	0.1	0.15	1.5	0.83	1.2	0.222	0.091	$-$0.015	$-$0.1
b	0.1	0.05	0.1	0.08	1.5	1.13	1.67	0.259	0.25	0.043	$-$0.03
c	0.18	0.05	0.06	0.05	2.3	1.1	2.8	0.407	0.474	0.307	0.009
d	0.09	0.05	0.06	0.05	1.4	1.1	1.6	0.221	0.231	0.1	$-$0.01

In the first scenario, it follows from Theorem 4.6 that $\mathcal {P}_2'(0+)\approx -0.015<0$ implies that $\mathcal {P}_2(\varepsilon )$ is strictly decreasing (see Fig. 1a). Thus, an increase in host dispersal reduces both disease prevalence and persistence. In the second scenario, the recovery rate $\gamma _2$ in scenario 1 is just lowered from 0.15 to 0.08. Then $\mathcal {P}'_2(0+)\approx 0.043>0$ and $\mathbb {P}_2'(\infty )=-0.03<0$ means that $\mathcal {P}_2(\varepsilon )$ is initially increasing then decreasing and there exists a unique critical point at $\varepsilon _0\approx 0.54$ corresponding to the absolute maximum of $\mathcal {P}_2(\varepsilon )$ (see Fig. 1b). Note that $\mathcal {P}_2(\infty )=0.25<\mathcal {P}_2(0)\approx 0.259$, so large dispersal results in fewer host infections compared to small or medium dispersal. In the third scenario, the facts $\mathcal {P}'_2(0+)\approx 0.307>0$ and $\mathbb {P}_2'(\infty )\approx 0.009>0$ imply that $\mathcal {P}_2(\varepsilon )$ is strictly increasing (see Fig. 1c). Thus, rapid dispersal is detrimental with respect to disease prevalence but beneficial with respect to disease persistence, showing an inconsistency between these two measures. In the last scenario, the transmission rate $\beta _1$ in scenario 3 is reduced from 0.18 to 0.09. Then $\mathcal {P}'_2(0+)\approx 0.1>0$ but $\mathbb {P}_2'(\infty )\approx -0.01<0$ imply that $\mathcal {P}_2(\varepsilon )$ is initially increasing then decreasing and there is exactly one critical point at $\varepsilon _0\approx 1.44$ corresponding to the absolute maximum of $\mathcal {P}_2(\varepsilon )$ (see Fig. 1d). Unlike scenario 2, dispersal consistently causes more host infections than no dispersal in scenario 4 due to $\mathcal {P}_2(\infty )\approx 0.231>\mathcal {P}_2(0)\approx 0.221$. It is worth mentioning that we can conclude that $\mathcal {P}_2(\infty )<\mathcal {P}_2(0)$ in the first two scenarios and $\mathcal {P}_2(\infty )>\mathcal {P}_2(0)$ in the last two scenarios by applying cases (a) and (b) of Proposition 3.10, respectively. The local host disease prevalence of patch 1 is strictly decreasing in all four scenarios while that of patch 2 is strictly increasing in scenarios 2–4 but initially increasing and then decreasing in the first scenario.

**Fig. 2:**
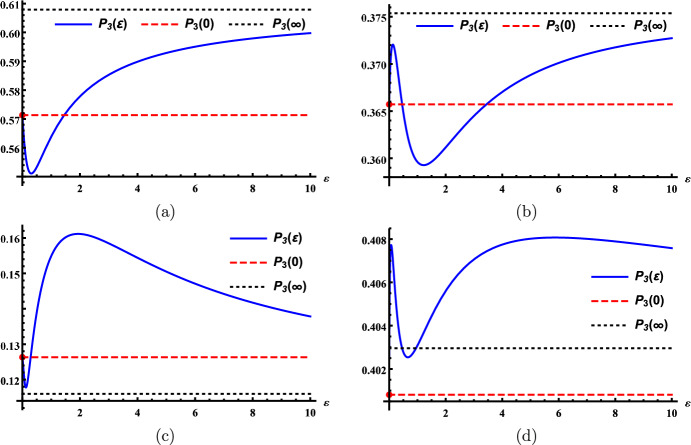
The global host disease prevalence $\mathcal {P}_3$ (blue solid line) versus host dispersal rate $\varepsilon$ under parameter settings **a** $\beta _1=0.26,$ $\gamma _1=0.04,$  $\beta _2=0.04,$ $\gamma _2=0.01,$ $\beta _3=0.03,$ $\gamma _3=0.08,$ $L_{12}=0.024,$ $L_{13}=0.04,$ $L_{21}=0.006,$ $L_{23}=0.08,$ $L_{31}=0.024,$ $L_{32}=0.02,$ **b** $\beta _1=0.088,$ $\beta _2=0.297,$ $\gamma _2=0.08,$ $\beta _3=0.04,$ $\gamma _3=0.068,$ $L_{12}=0.007,$ $L_{13}=0.033,$ $L_{21}=0.023,$ $L_{23}=0.0045,$ $L_{31}=0.038,$ $L_{32}=0.024,$ **c** $\beta _1=0.263,$ $\gamma _1=0.08,$ $\beta _2=0.04,$ $\gamma _2=0.019,$ $\beta _3=0.021,$ $\gamma _3=0.043,$ $L_{12}=0.039,$ $L_{13}=0.0033,$ $L_{21}=0.006,$ $L_{23}=0.03,$ $L_{31}=0.06,$ $L_{32}=0.099,$ and **d** $\beta _1=0.045,$ $\gamma _1=0.011,$ $\beta _2=0.291,$ $\gamma _2=0.077,$ $\beta _3=0.087,$ $\gamma _3=0.095,$ $L_{12}=0.027,$ $L_{13}=0.024,$ $L_{21}=0.02,$ $L_{23}=0.01,$ $L_{31}=0.032,$ $L_{32}=0.0015;$ and $\sigma _i=1$ for $i=1,2,3$ in all four scenarios. The red dot on the left *y*-axis corresponds to the point $(0,\mathcal {P}_3(0)).$

When the patchy environment consists of $n\ge 3$ patches, the global host disease prevalence $\mathcal {P}_n(\varepsilon )$ exhibits more changing patterns. For example, in the three-patch case, we observe new patterns such as initially decreasing and then increasing, initially increasing and then decreasing followed by eventually increasing, initially decreasing and then increasing followed by eventually decreasing, and initially increasing then decreasing then increasing and eventually decreasing (see Fig. 2). In other words, $\mathcal {P}_n(\varepsilon )-\mathcal {P}_n(0)$ and $\mathcal {P}_n'(\varepsilon )$ can change signs multiple times when $n\ge 3$, and at most once when there are two patches.

### Example 5.2

(Host and vector prevalences) Vector-borne diseases are transmitted back and forth between vectors and hosts. In a disconnected patch, an increase in host disease prevalence leads to a larger proportion of mosquito bites on infected hosts, which in turn results in more vector infections and an increase in vector disease prevalence. Conversely, an increase in vector disease prevalence results in more infectious bites received by each host, causing more host infections and an increase in host disease prevalence. Thus, we may expect that the host and vector disease prevalences change synchronously in response to any parameter change. As noted in Remark 3.4 and Corollary 3.3, this synchrony holds true for both single-patch and multi-patch cases with respect to non-travel-related parameters.

**Fig. 3:**
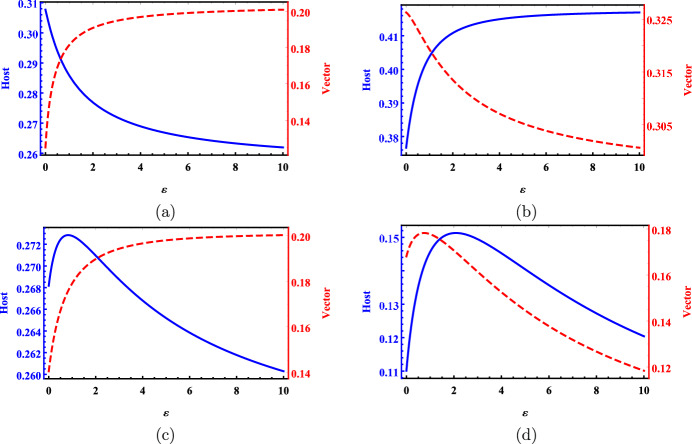
The global host and vector disease prevalences, $\mathcal {P}_2$ (blue solid line) and $\mathscr {P}_2$ (red dashed line), versus host dispersal rate $\varepsilon$ under parameter settings **a** $\beta _1=\beta _2=0.034,$ $\gamma _1=0.017,$ $\gamma _2=0.058,$ $L_{12}=0.036, L_{21}=0.003,$ **b** $\beta _1=0.212,$ $\beta _2=0.079,$ $\gamma _1=\gamma _2=0.04,$ $L_{12}=0.008,$ $L_{21}=0.05,$ **c** $\beta _1=0.226,$ $\beta _2=0.033,$ $\gamma _1=\gamma _2=0.099,$ $L_{12}=0.035,$ $L_{21}=0.016,$ and **d** $\beta _1=0.29,$ $\beta _2=0.06,$ $\gamma _1=\gamma _2=0.095,$ $L_{12}=0.01,$ $L_{21}=0.036;$ and $\sigma _1=\sigma _2=1$ and $V_1=V_2=0.5$ in all four scenarios. The *x*-axis is for $\varepsilon$ while the left and right *y*-axes represent $\mathcal {P}_2$ and $\mathscr {P}_2,$ respectively.

However, the relationship between host and vector disease prevalences with respect to host dispersal rate may not always be consistent, as illustrated in Fig. 3. In panels a and b , the changing patterns of host and vector disease prevalences are opposite. In panel c , their monotonicities are the same under small dispersal but differ under large dispersal. In panel d , both prevalences have the same changing patterns, but they reach their maximum values at different dispersal rates. This suggests that changes in vector disease prevalence cannot reliably be used to predict changes in host disease prevalence when these changes are attributed to host movement.

### Example 5.3

(Nonhomogeneous mixing) Host movement is characterized by dispersal rate $\varepsilon$ and patch connectivity matrix *L*. The role of habitat connectivity on disease prevalence is not well understood, primarily because *L* depends on $n^2-n$ parameters for an *n*-patch environment. Let us consider a two-patch homogenous environment where the two patches have identical parameter settings when disconnected, i.e.,

$$\begin{aligned} a_i=a,\quad b_i=b,\quad c_i=c,\quad \gamma _i=\gamma ,\quad \mu _i=\mu ,\quad V_i=V_0/2,\quad i=1,2. \end{aligned}$$

For convenience, we set $a=b=c=\mu =\gamma =H=\varepsilon =1$. Then the equilibrium equations (4.2) become

$$\begin{aligned} \begin{aligned} \frac{V_0}{2\alpha _i}\left( 1-\frac{2I_i^*}{I_i^*+\alpha _i }\right) I_i^*-I_i^*+\sum \limits _{j=1}^2L_{ij}I_j^*=0, \quad 1\le i\le 2, \end{aligned} \end{aligned}$$

and the associated basic reproduction number is $\mathcal {R}_0=\rho (FV^{-1})$ where

$$F=\begin{pmatrix} \frac{V_0}{2\alpha _1} & \quad 0 \\ 0 & \quad \frac{V_0}{2\alpha _2} \end{pmatrix} \quad \text{ and }\quad V=\begin{pmatrix} 1 & \quad 0 \\ 0 & \quad 1 \end{pmatrix}-\begin{pmatrix} -L_{21} & \quad L_{12} \\ L_{21} & \quad L_{21} \end{pmatrix}.$$

In particular, if the dispersal is symmetric, i.e., $L_{12}=L_{21}$, then the host and vector disease prevalences, and the basic reproduction number are respectively

5.1 $$\begin{aligned} \begin{aligned}&\left( 1-\frac{2}{V_0+1}\right) ^+,\quad \frac{1}{2}\left( 1-\frac{1}{V_0}\right) ^+,\quad \text{ and }\quad \mathcal {R}_0=V_0. \end{aligned} \end{aligned}$$

**Fig. 4:**
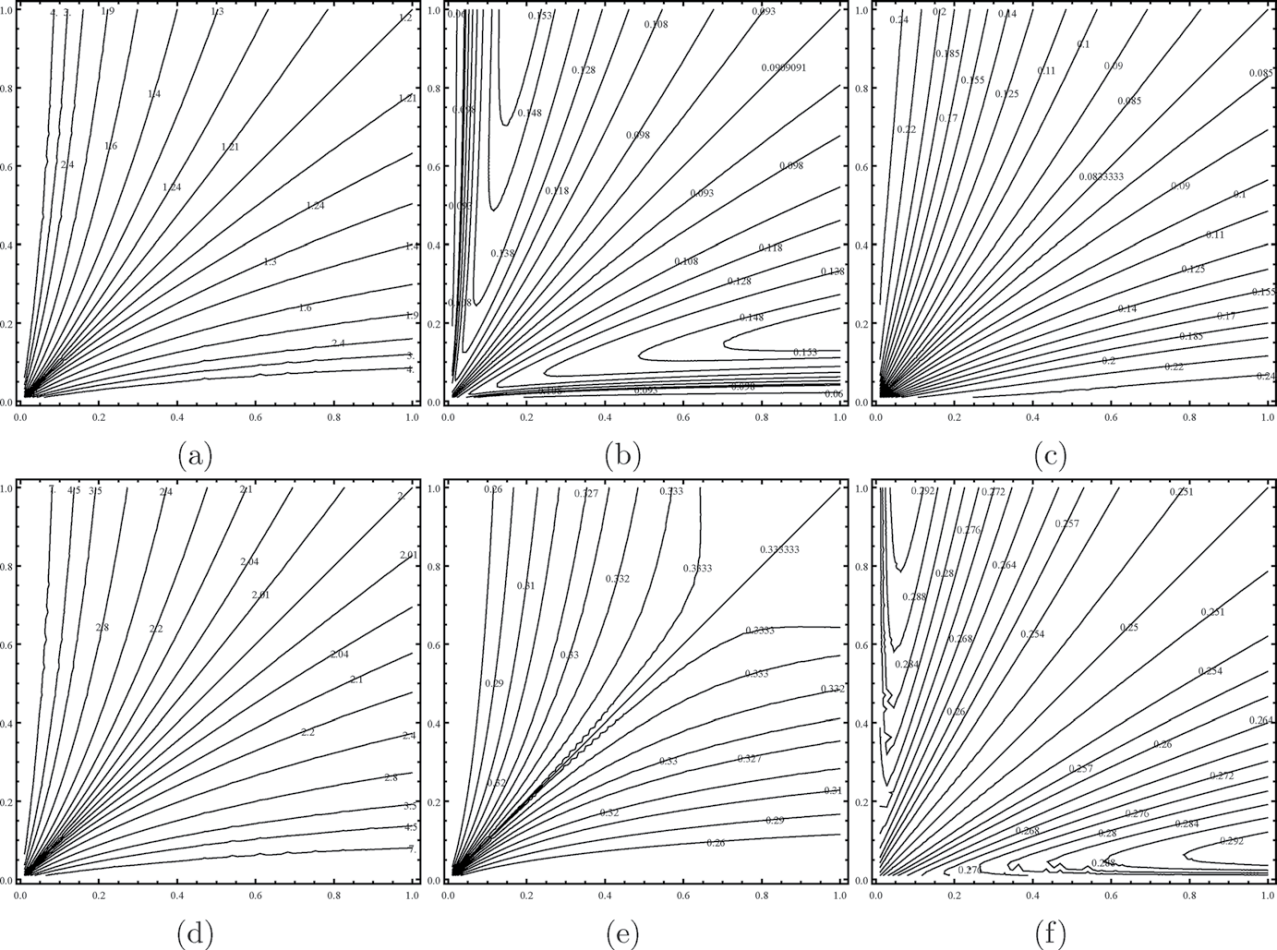
The contour plots of the basic reproduction number $\mathcal {R}_0$ (first column), the global host disease prevalence (second column), and the global vector disease prevalence (third column) versus $L_{12}$ and $L_{21}$ under small vector abundance (first row) and large vector abundance (second row). The *x* and *y*-axes are for $L_{12}$ and $L_{21}$, respectively. See text for parameter settings.

One can also verify that the disease prevalences and the reproduction number tend to the above values when $L_{12}\rightarrow \infty$ and $L_{12}$ and $L_{21}$ are proportional (same as $\varepsilon \rightarrow \infty$ for fixed *L*). We plot the contour plots of the basic reproduction number $\mathcal {R}_0$, the global host disease prevalence $\mathcal {P}_2$, and the global vector disease prevalence $\mathscr {P}_2$ as functions of $L_{12}$ and $L_{21}$ under small vector abundance $V_0=1.2$ (Fig. 4a–c) and large vector abundance $V_0=2$ (Fig. 4d–f). In both cases, it follows from Proposition 2.2 that the minimum of $\mathcal {R}_0$ is $V_0$ and $\mathcal {R}_0=V_0$ if and only if the vector and host populations are uniformly distributed, meaning $L_{12}=L_{21}$. In other words, nonhomogeneous mixing of hosts and vectors in a homogeneous environment always increases $\mathcal {R}_0$ (Gao et al. 2019). Moreover, for fixed $L_{ij}>0$, we observe that $\mathcal {R}_0\rightarrow \infty$ as $L_{ji}\rightarrow 0+$ and $\mathcal {R}_0$ is initially decreasing then increasing in $L_{ji}$.

In the case of small vector abundance, nonhomogeneous mixing of hosts and vectors generally enhances both host and vector disease prevalences. This is not surprising, as the disease approaches extinction when $\mathcal {R}_0$ is very close to 1. Moreover, for fixed $L_{ij}>0$, the host prevalence $\mathcal {P}_2$ is initially increasing and then decreasing (or followed by increasing, or followed by increasing and eventually decreasing) in $L_{ji}$ while the vector prevalence $\mathscr {P}_2$ is initially decreasing (or initially increasing and then decreasing) and then increasing in $L_{ji}$. However, in the case of large vector abundance, nonhomogeneous mixing of hosts and vectors generally reduces host disease prevalence but still enhances vector disease prevalence. Moreover, for fixed $L_{ij}>0$, the host prevalence $\mathcal {P}_2$ is initially increasing and then decreasing followed by increasing (or eventually decreasing) in $L_{ji}$ while the vector prevalence $\mathscr {P}_2$ is initially decreasing (or initially increasing and then decreasing) and then increasing in $L_{ji}$. Thus, even within a two-patch homogeneous environment, the relationship between disease prevalences and habitat connectivity is complex. Interestingly, nonhomogeneous mixing of hosts and vectors has a high probability of increasing disease persistence and vector disease prevalence but decreasing host disease prevalence.

## Discussion

For vector-borne diseases, host and vector movements are important in global and local transmission, respectively. Understanding their roles in disease spread is crucial for designing effective control measures, such as travel restrictions and vector management strategies. A variety of spatial vector-borne disease models have been developed and studied, with a primary emphasis on dynamic analysis. The persistence of diseases like West Nile virus in the United States since its introduction into New York City in 1999 and Zika virus in Brazil since the 2015–2016 Zika virus epidemic, as well as the re-emergence of locally acquired malaria in the United States, underscores the challenges of eliminating vector-borne diseases. Consequently, it is of significant epidemiological importance to study how population mobility affects the total number of host infections and its spatial distribution within endemic regions.

In this paper, using a multi-patch Ross–Macdonald model, we studied the influence of host migration on local and global host disease prevalences. We first reduced the number of equilibrium equations which enables us to write disease prevalences in a simple way. Then we estimated the local disease prevalence of any connected patch and found that it lies between the minimum and maximum disease prevalences of all disconnected patches. Additionally, we discovered a weak order-preserving property for the *n*-patch environment, which becomes strong when $n=2$. For global disease prevalence, we derived its formula at zero and infinite dispersals and compared them under three biologically meaningful conditions, and computed the right derivative at zero dispersal. To get better results, our attention is limited to the two-patch case. Two key lemmas were proved and used to give two complete classifications of model parameter space: one is to compare $\mathcal {P}_2(\varepsilon )$ and $\mathcal {P}_2(0)$, the other is on the monotonicity of $\mathcal {P}_2(\varepsilon )$. The global host disease prevalence is either strictly decreasing, or strictly increasing, or initially strictly increasing and then strictly decreasing, or constant with respect to host dispersal rate. In addition, the local disease prevalence of the high-risk patch is strictly decreasing while that of the low-risk patch is either strictly increasing or initially strictly increasing and then strictly decreasing as long as the disease persists.

Finally, we presented three numerical examples to further investigate the impact of host movement on disease prevalence. In the first example, we demonstrated the two classifications for the two-patch case, indicating that large dispersal tends to result in more host infections compared to small or no dispersal. For three-patch case, the global host disease prevalence $\mathcal {P}_3(\varepsilon )$ exhibits up to eight distinct changing patterns and three critical points, suggesting the greater difficulty in doing similar classifications. In the second example, we observed that the responses of hosts and vectors to fast host dispersal can be partially or completely inconsistent. This suggests that using vector infections to assess the effectiveness of travel interventions may not provide reliable results. The last example concentrates on the effects of nonhomogeneous mixing of hosts and vectors in a homogeneous environment. In general, we found that the more uneven the distribution of hosts and vectors, the lower the prevalence of the host, but the higher the prevalence of the vector. In other words, hosts and vectors often respond differently to their spatial mixing.

To our knowledge, this is the first theoretical study to explore the impact of host movement on both local and global host disease prevalences. While it has some similarities with our previous work on the SIS patch model (Gao 2020; Gao and Lou 2021), it also presents significant differences. Unlike directly transmitted diseases, vector-borne diseases involves both hosts and vectors. We are mainly interested in the host disease prevalence, as there is no joint prevalence between hosts and vectors. The patch reproduction number $\mathcal {R}_0^{(i)}$ determines whether a vector-borne disease can spread within a disconnected patch *i*. However, unlike the SIS model, $\mathcal {R}_0^{(i)}$ cannot be used to characterize the host disease prevalence. This difference inspires us to introduce the host patch reproduction number $\mathcal {R}_h^{(i)}$ such that $(1-1/\mathcal {R}_h^{(i)})^+$ is the host disease prevalence of patch *i* in disconnection. The host patch reproduction number plays a vital role in model analysis, such as in defining high/low-risk patch and in excluding ideal free distribution strategy. The global host disease prevalence $\mathcal {P}_n(\varepsilon )$ is constant if $\mathcal {R}_h^{(i)}$ (not $\mathcal {R}_0^{(i)}$) is constant in $i\in \Omega$. In contrast to the method used in Gao and Lou (2021) to prove the monotonicity of $\mathcal {P}_2(\varepsilon )$ under $\mathbb {P}_2'(\infty )=0$, the approach here is simpler and more effective for the current model, relying on the monotonicity of local host disease prevalence. One particularly fascinating result involves the effects of nonhomogeneous mixing. In the SIS patch model, nonhomogeneous mixing of the susceptible and infectious individuals (caused by different connectivity matrices for susceptible and infectious populations) in a homogeneous environment does not affect disease persistence (Gao and Ruan 2011), but it always reduces disease prevalence (Gao and Li 2025). However, in the multi-patch Ross–Macdonald model, nonhomogeneous mixing of hosts and vectors (due to different connectivity matrices for host and vector populations) in a homogeneous environment always promotes disease persistence (Gao et al. 2019), but it probably reduces host disease prevalence and enhances vector disease prevalence. Similar phenomena have been partially observed in Lagrangian and hybrid Lagrangian–Eulerian vector-borne disease models (Gao and Cao 2024; Gao and Yuan 2024). A plausible explanation is that, while the nonhomogeneous mixing of hosts and vectors does not alter the total number of bites, it causes a significant proportion of bites to be concentrated on a few hosts. As a result, the vast majority of hosts are spared, ultimately reducing the overall host infection size.

There is much room for improvement and generalization. The upper and lower bounds on local disease prevalence may be too broad to provide useful insights, especially when the environment consists of a large number of distinct patches. The two complete classifications on global host disease prevalence apply only to the two-patch case. When considering three or more patches environment, $\mathcal {P}_n(\varepsilon )$ can have multiple critical points and richer behaviors, as illustrated in Fig. 2, suggesting the conclusion and analytical approach of the two key lemmas, Lemmas 4.1 and 4.4, are no longer valid. We want to give an intuitive interpretation of these classification conditions and figure out which scenario is most likely to occur in reality. In the two-patch SIS model, increasing movement from a high-risk patch to a low-risk patch reduces both disease persistence and the local/global disease prevalence (Gao and Wang 2024). However, the dependences of disease persistence and prevalence on dispersal asymmetry for our current model are more intricate even in a two-patch homogenous environment (see Fig. 4). What are the effects of nonhomogeneous mixing of hosts and vectors in a heterogeneous environment? In particular, are there lower and upper bounds on the prevalence (dependent or independent of *L*) that are not equal to 0 or 1? Note that the estimate given in Proposition 3.6 depends on *L*. The role of habitat connectivity on disease prevalence deserves a detailed study. Can we define a multi-patch host or vector reproduction number, $\mathcal {R}_h$ or $\mathcal {R}_v$? How to interpret these single- and multi-patch host/vector reproduction numbers biologically? Mathematically, it would be interesting to study how local and global vector disease prevalence change with host movement. For certain vector-borne diseases, such as malaria, infection can substantially reduce the mobility of infected individuals. Therefore, it is natural to assume that susceptible and infected hosts have different dispersal rates or connectivity matrices. In this context, we are particularly interested in the asymptotic profiles of the endemic equilibrium as the host dispersal rate approaches zero (Allen et al. 2007). The Ross–Macdonald patch model proposed by Auger et al. (2008) assumes that some patches are free of vectors. Additionally, we ignored vector movement and considered Eulerian movement for hosts. What if vectors also move or hosts follow Lagrangian movement (Gao and Cao 2024; Gao and Yuan 2024)? or local or nonlocal diffusion in continuous space (Magal et al. 2018)? or return-to-home process (Ducrot and Magal 2022; Magal 2024)? The Ross–Macdonald model is the simplest vector-borne disease model. We would like to extend the current study to more realistic models by incorporating more biological and epidemiological factors in the future.

## Data Availability

This manuscript has no associated data.

## Appendix A: Proof of Theorem 3.8

### Proof

The case of $\varepsilon \rightarrow 0+$ can be easily obtained from (3.6). Suppose $\mathcal {R}_0(\infty )>1$. It follows from $\varvec{I}^*(\varepsilon )\in \Gamma$ that there exists some $\tau _n\in [0,1]$ such that (up to a sequence of $\varepsilon$)

$$\lim _{\varepsilon \rightarrow \infty }(I_1^*(\varepsilon ),\dots ,I_n^*(\varepsilon )) =\tau _n(\alpha _1,\dots ,\alpha _n).$$

Summing up (3.2) over $i\in \Omega$ yields

$$\begin{aligned} \begin{aligned} \sum _{i\in \Omega }\left( \beta _i-\frac{\sigma _i+1}{\sigma _i I_i^*(\varepsilon )+\alpha _i}\beta _iI_i^*(\varepsilon )-\gamma _i\right) I_i^*(\varepsilon )=0. \end{aligned} \end{aligned}$$

As $\varepsilon \rightarrow \infty$ (up to a sequence), we have

$$\sum _{i\in \Omega }\left( \beta _i-\frac{\sigma _i+1}{\sigma _i \tau _n\alpha _i+\alpha _i}\beta _i\tau _n\alpha _i -\gamma _i\right) \tau _n\alpha _i=0.$$

It follows from a similar argument as Theorem 3.6 in Gao and Dong (2020) and the estimate on $\mathcal {P}_n(\varepsilon )$ in Proposition 3.6 that $\tau _n\ne 0$ or 1. Thus, the above equation can be simplified to

$$\sum _{i\in \Omega }\left( \beta _i-\frac{\sigma _i+1}{\sigma _i \tau _n+1}\beta _i\tau _n-\gamma _i\right) \alpha _i =\sum _{i\in \Omega }\left( \beta _i\frac{1-\tau _n}{\sigma _i\tau _n+1}-\gamma _i\right) \alpha _i=0.$$

Denote the left-hand side of the above by $p(\tau _n)$, a function of $\tau _n$. Direct calculations give

$$\begin{aligned} \begin{aligned} p\left( 1-\frac{1}{\mathcal {R}_0(\infty )}\right)&=\sum _{i\in \Omega }\left( \beta _i\frac{1-(1-1/\mathcal {R}_0(\infty ))}{\sigma _i(1-1/\mathcal {R}_0(\infty ))+1}-\gamma _i\right) \alpha _i \\&=\sum _{i\in \Omega }\left( \frac{\beta _i}{\sigma _i(\mathcal {R}_0(\infty )-1)+\mathcal {R}_0(\infty )}-\gamma _i\right) \alpha _i \\&<\sum _{i\in \Omega }\left( \frac{\beta _i}{\mathcal {R}_0(\infty )}-\gamma _i\right) \alpha _i\\&=\frac{1}{\mathcal {R}_0(\infty )}\sum _{i\in \Omega }\beta _i\alpha _i-\sum _{i\in \Omega }\gamma _i\alpha _i=0 \end{aligned} \end{aligned}$$

and

$$\begin{aligned}&p(0)=\sum _{i\in \Omega }(\beta _i-\gamma _i)\alpha _i>0\ \text{ and } \\&p'(x)=-\sum _{i\in \Omega }\beta _i\alpha _i\frac{\sigma _i+1}{(\sigma _i x+1)^2}<0,\ x\in (0,1). \end{aligned}$$

So, by the Intermediate Value Theorem and the monotonicity of *p*(*x*), there exists a unique $\tau _n\in (0,1-1/\mathcal {R}_0(\infty ))$ such that $p(\tau _n)=0$. $\square$

## Appendix B: Proof of Proposition 3.10

### Proof

Suppose $\mathcal {R}_0^{(i)}>1$, or equivalently, $\mathcal {R}_h^{(i)}>1$, and $\sigma _i=\sigma$. Then

$$p(\mathcal {P}_n(0))=\sum _{i\in \Omega }\left( \beta _i\frac{1-\mathcal {P}_n(0)}{\sigma \mathcal {P}_n(0)+1}-\gamma _i\right) \alpha _i =\frac{1-\mathcal {P}_n(0)}{\sigma \mathcal {P}_n(0)+1}\sum _{i\in \Omega }\beta _i\alpha _i-\sum _{i\in \Omega }\gamma _i\alpha _i,$$

where $p(\cdot )$ is defined in the proof of Theorem 3.8 satisfying $p'(x)<0$ and $p(\mathcal {P}_n(\infty ))=0$, and

$$\mathcal {P}_n(0)=\sum _{j\in \Omega }\left( 1-\frac{1}{\mathcal {R}_h^{(j)}}\right) \alpha _j =1-\sum _{j\in \Omega }\frac{\alpha _j}{\mathcal {R}_h^{(j)}} =1-\sum _{j\in \Omega }\frac{(1+\sigma )\gamma _j}{\beta _j+\sigma \gamma _j}\alpha _j.$$

Case (a): $\beta _i=\beta$. It suffices to check that $p(\mathcal {P}_n(0))\le 0$, i.e.,

$$\begin{aligned} \begin{aligned}&p(\mathcal {P}_n(0))=\frac{1-\mathcal {P}_n(0)}{\sigma \mathcal {P}_n(0)+1} \beta -\sum _{i\in \Omega }\gamma _i\alpha _i\le 0 \\&\quad \Leftrightarrow \, \mathcal {P}_n(0)=1-\sum _{j\in \Omega }\frac{(1+\sigma )\gamma _j}{\beta +\sigma \gamma _j}\alpha _j \ge \frac{\beta -\sum _{i\in \Omega }\gamma _i\alpha _i}{\beta +\sigma \sum _{i\in \Omega }\gamma _i\alpha _i} \\&\quad \Leftrightarrow \, 1-\frac{\beta -\sum _{i\in \Omega }\gamma _i\alpha _i}{\beta +\sigma \sum _{i\in \Omega }\gamma _i\alpha _i} =(1+\sigma )\frac{\sum _{i\in \Omega }\gamma _i\alpha _i}{\beta +\sigma \sum _{i\in \Omega }\gamma _i\alpha _i}\\&\qquad \ge (1+\sigma )\sum _{j\in \Omega }\frac{\gamma _j\alpha _j}{\beta +\sigma \gamma _j}\\&\quad \Leftrightarrow \, \sum _{i\in \Omega }\gamma _i\alpha _i \ge \left( \beta +\sigma \sum _{i\in \Omega }\gamma _i\alpha _i\right) \sum _{j\in \Omega }\frac{\gamma _j\alpha _j}{\beta +\sigma \gamma _j}. \end{aligned} \end{aligned}$$

By the Cauchy–Schwarz inequality, we have

$$\begin{aligned} \begin{aligned}&\left( \beta +\sigma \sum _{i\in \Omega }\gamma _i\alpha _i\right) \sum _{j\in \Omega }\frac{\gamma _j\alpha _j}{\beta +\sigma \gamma _j}\\=&\left( \beta +\sigma \sum _{i\in \Omega }\gamma _i\alpha _i\right) \frac{1}{\sigma } \sum _{j\in \Omega }\frac{\sigma \gamma _j+\beta -\beta }{\beta +\sigma \gamma _j}\alpha _j\\=&\frac{1}{\sigma }\left( \beta +\sigma \sum _{i\in \Omega }\gamma _i\alpha _i\right) \left( 1-\beta \sum _{j\in \Omega } \frac{\alpha _j}{\beta +\sigma \gamma _j}\right) \\=&\frac{1}{\sigma }\left( \beta +\sigma \sum _{i\in \Omega }\gamma _i\alpha _i -\beta \sum _{i\in \Omega }(\beta +\sigma \gamma _i)\alpha _i\cdot \sum _{j\in \Omega } \frac{\alpha _j}{\beta +\sigma \gamma _j}\right) \\ \le& \frac{1}{\sigma }\left( \beta +\sigma \sum _{i\in \Omega }\gamma _i\alpha _i -\beta \left( \sum _{i\in \Omega }\alpha _i\right) ^2\right) \\=&\frac{1}{\sigma }\left( \beta +\sigma \sum _{i\in \Omega }\gamma _i\alpha _i -\beta \right) =\sum _{i\in \Omega }\gamma _i\alpha _i. \end{aligned} \end{aligned}$$

Case (b): $\gamma _i=\gamma$. It suffices to verify that $p(\mathcal {P}_n(0))\ge 0$, i.e.,

$$\begin{aligned} \begin{aligned}&p(\mathcal {P}_n(0))=\frac{1-\mathcal {P}_n(0)}{\sigma \mathcal {P}_n(0)+1}\sum _{i\in \Omega }\beta _i\alpha _i -\gamma \ge 0 \\&\quad \Leftrightarrow \ \mathcal {P}_n(0)=1-\sum _{j\in \Omega }\frac{(1+\sigma )\gamma }{\beta _j+\sigma \gamma }\alpha _j \le \frac{\sum _{i\in \Omega }\beta _i\alpha _i-\gamma }{\sum _{i\in \Omega }\beta _i\alpha _i+\sigma \gamma } \\&\quad \Leftrightarrow \ (1+\sigma )\gamma \sum _{j\in \Omega }\frac{\alpha _j}{\beta _j+\sigma \gamma }\ge 1-\frac{\sum _{i\in \Omega }\beta _i\alpha _i-\gamma }{\sum _{i\in \Omega }\beta _i\alpha _i+\sigma \gamma }\\&\qquad = \frac{(1+\sigma )\gamma }{\sum _{i\in \Omega }\beta _i\alpha _i+\sigma \gamma }\\&\quad \Leftrightarrow \ \sum _{j\in \Omega }\frac{\alpha _j}{\beta _j+\sigma \gamma }\cdot \sum _{i\in \Omega }(\beta _i+\sigma \gamma )\alpha _i\ge 1=\left( \sum _{i\in \Omega }\sqrt{\alpha _i^2}\right) ^2. \end{aligned} \end{aligned}$$

The last step is again due to the Cauchy–Schwarz inequality.

Case (c): $\beta _i-\gamma _i=c>0$. It suffices to verify that $p(\mathcal {P}_n(0))\le 0$, i.e.,

$$\begin{aligned} \begin{aligned}&p(\mathcal {P}_n(0))=\left( \frac{1-\mathcal {P}_n(0)}{\sigma \mathcal {P}_n(0)+1}-1\right) \sum _{i\in \Omega }\beta _i\alpha _i+c\le 0 \\&\quad \Leftrightarrow \ \mathcal {P}_n(0)=1-\sum _{j\in \Omega }\frac{(1+\sigma )\beta _j-(1+\sigma )c}{(1+\sigma )\beta _j-\sigma c}\alpha _j\\&\qquad = \sum _{j\in \Omega }\frac{c}{(1+\sigma )\beta _j-\sigma c}\alpha _j \\&\qquad \ \ge \frac{c}{(1+\sigma ) \sum _{i\in \Omega }\beta _i\alpha _i-c\sigma }=\frac{c}{\sum _{i\in \Omega } ((1+\sigma )\beta _i-\sigma c)\alpha _i}\\&\quad \Leftrightarrow \ \sum _{i\in \Omega } ((1+\sigma )\beta _i-\sigma c)\alpha _i\cdot \sum _{j\in \Omega }\frac{\alpha _j}{(1+\sigma )\beta _j-\sigma c}\ge 1. \end{aligned} \end{aligned}$$

The final step follows once more from the Cauchy–Schwarz inequality. $\square$
